# The unprecedented Pacific Northwest heatwave of June 2021

**DOI:** 10.1038/s41467-023-36289-3

**Published:** 2023-02-09

**Authors:** Rachel H. White, Sam Anderson, James F. Booth, Ginni Braich, Christina Draeger, Cuiyi Fei, Christopher D. G. Harley, Sarah B. Henderson, Matthias Jakob, Carie-Ann Lau, Lualawi Mareshet Admasu, Veeshan Narinesingh, Christopher Rodell, Eliott Roocroft, Kate R. Weinberger, Greg West

**Affiliations:** 1 Department of Earth, Ocean and Atmospheric Sciences, University of British Columbia, Vancouver, BC Canada; 2 Earth and Atmospheric Science, City College of New York, New York, NY US; 3 The Graduate Center, City University of New York, New York, NY US; 4 Institute for Resources, Environment and Sustainability, University of British Columbia, Vancouver, BC Canada; 5 Department of Zoology, University of British Columbia, Vancouver, BC Canada; 6 Environmental Health Services, British Columbia Centre for Disease Control (BCCDC), Vancouver, BC Canada; 7 School of Population and Public Health, University of British Columbia, Vancouver, BC Canada; 8 BCG Engineering Inc, Vancouver, BC Canada; 9 NOAA Geophysical Fluid Dynamics Laboratory, Program in Atmosphere and Ocean Sciences, Princeton University, Princeton, NJ US; 10 BC Hydro, Vancouver, BC Canada

**Keywords:** Natural hazards, Climate sciences, Climate-change impacts

## Abstract

In late June 2021 a heatwave of unprecedented magnitude impacted the Pacific Northwest region of Canada and the United States. Many locations broke all-time maximum temperature records by more than 5 °C, and the Canadian national temperature record was broken by 4.6 °C, with a new record temperature of 49.6 °C. Here, we provide a comprehensive summary of this event and its impacts. Upstream diabatic heating played a key role in the magnitude of this anomaly. Weather forecasts provided advanced notice of the event, while sub-seasonal forecasts showed an increased likelihood of a heat extreme with lead times of 10-20 days. The impacts of this event were catastrophic, including hundreds of attributable deaths across the Pacific Northwest, mass-mortalities of marine life, reduced crop and fruit yields, river flooding from rapid snow and glacier melt, and a substantial increase in wildfires—the latter contributing to landslides in the months following. These impacts provide examples we can learn from and a vivid depiction of how climate change can be so devastating.

## Abstract (web summary)

The 2021 unprecedented Pacific Northwest heatwave broke temperature records by extraordinary amounts. Impacts included hundreds of deaths, mass-mortalities of marine life, increased wildfires, reduced crop and fruit yields, and river flooding.

## Introduction

**Fig. 1:**
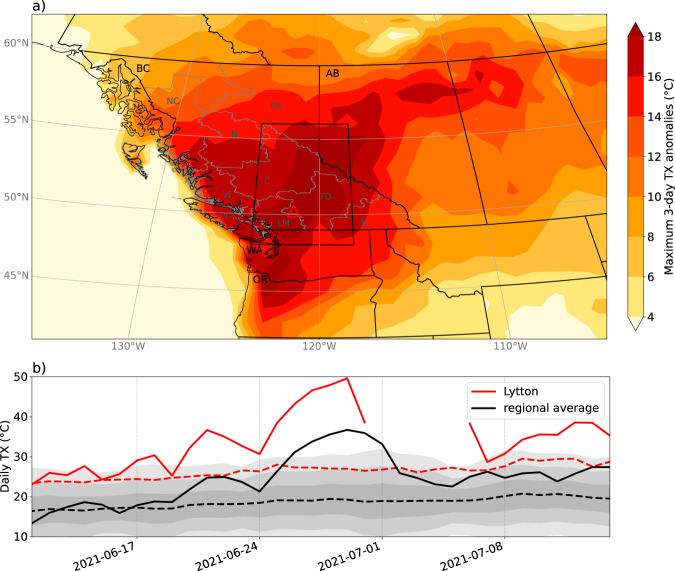
June 2021 Pacific Northwest heatwave temperatures. Heatwave daily maximum near-surface (2 m) temperatures (TX). **a** ERA5 reanalysis data maximum 3-day running mean (between 23 June and 02 July 2021) of TX anomalies relative to a daily 1981–2020 climatology (see Methods for more details). **b** Absolute TX values for 2021 (solid) and 1981–2020 climatology (dashed); black lines: spatial average of ERA5 data over the black box in **a**, with shading ±1, 2, and 3 standard deviations; red lines: observations from Lytton, British Columbia (BC), denoted by the red triangle in **a**—missing values from 1 to 5 July are likely due to the wildfire. Black letters and outlines in **a** show the main Canadian provinces and US states referred to in this study. Gray outlines and letters in **a** show the eight crop regions in BC, discussed further in the section on Agricultural Yields.

**Fig. 2:**
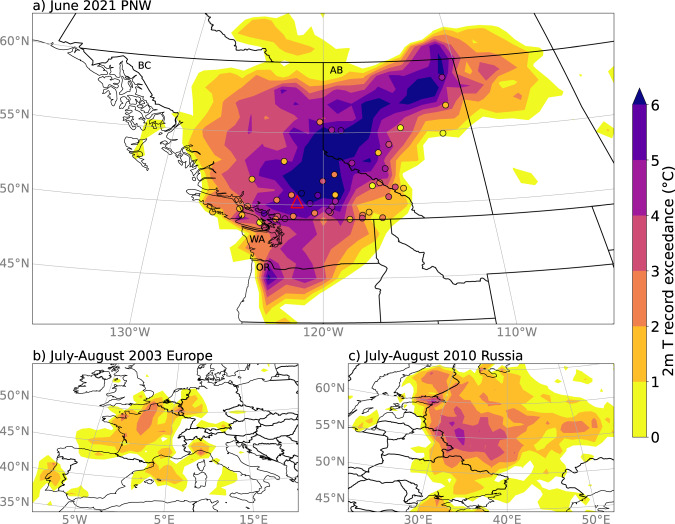
Temperature record exceedances. Exceedance of previous record high temperatures during **a** the June 2021 Pacific Northwest heatwave, **b** the July–August European heatwave of 2003, and **c** the July–August Russian heatwave of 2010. Filled contours show ERA5 since 1950, whilst individual markers show observational station data in Canada; see Methods for record lengths.

An unprecedented heatwave occurred in the Pacific Northwest (PNW) from ~25 June to 2 July 2021, over lands colonially named British Columbia (BC) and Alberta (AB) in Canada, Washington (WA), and Oregon (OR) in the United States. Near-surface air temperature anomalies reached up to 16–20 °C above normal over a wide region (Fig. 1), with many locations breaking all-time maximum temperature records by more than 5 °C (Fig. 2a). The Canadian national temperature record was broken 3 days in a row, at multiple locations, with the highest temperature of 49.6 °C recorded in Lytton, BC, on 29 June (Figs. 1b), 4.6 °C higher than the Canadian record prior to this event. Lytton, a small town in an arid mountain valley just north of 50°N in the lee of the BC Coast Range (shown by the red triangle in Fig. 1a), is located on Lytton First Nation reserves, at the site of the Indigenous village of Kumsheen on the traditional lands of the Nlaka’pamux people. On 30 June 2021, much of Lytton was tragically destroyed by a wildfire. The new record temperature was reportedly the hottest worldwide temperature recorded north of 45° latitude, and hotter than any recorded temperature in Europe or South America^1^. The amount by which previous all-time records were broken was extraordinary when compared with the infamous heatwaves in Europe in August 2003 and Russia in July–August 2010 (Fig. 2), both of which killed 10,000 s of people^2,3^. Notably, whilst the record exceedance was much higher for this PNW heatwave, and the maximum anomalies in standard deviations were also higher^4^, the June 2021 PNW heatwave was shorter in duration than these previous two heatwaves.

## Results

### Synoptic conditions

**Fig. 3:**
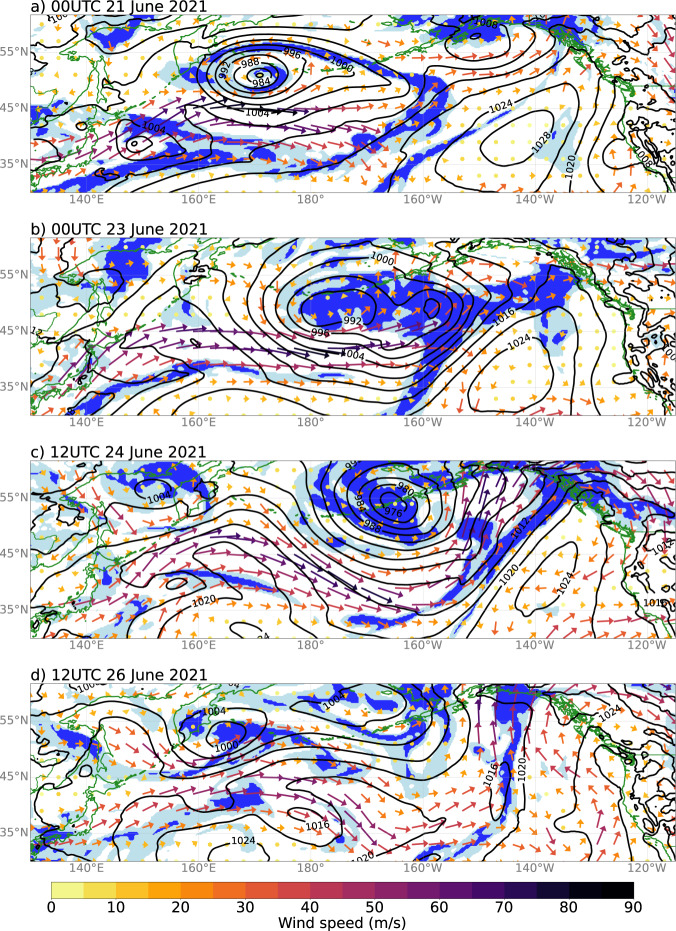
Meteorological conditions leading up to heatwave onset. Meteorological conditions over the North Pacific for **a** 00UTC 21 June, **b** 00UTC 23 June, **c** 12UTC 24 June, and **d** 12UTC 26 June. Data from ERA5 reanalysis showing: mean sea-level pressure (MSLP; contoured, hPa), 700 hPa relative humidity (RH; shaded, light blue >70%, dark blue >90%), and 250 hPa wind vectors (ms^−1^, colored by wind speed). Coastlines and country borders are shown in green to distinguish them from the MSLP contours.

The synoptic conditions leading to this event can be traced back about a week prior to the onset of the heatwave on 25 June (the date regional temperature anomalies first exceeded 2 standard deviations, see black line in Fig. 1b). Mean sea-level pressure, 700 hPa relative humidity and 250 hPa winds from 21 June to 26 June are shown in Fig. 3. From 19–21 June a low-pressure system intensified southeast of the Kamchatka Peninsula of eastern Russia (Fig. 3a, low centered at 50°N, 172°E); the associated northerly winds transported cold air southward over the northwest Pacific Ocean. Concurrently, southwesterly flow along and within a frontal zone (denoted by high relative humidity values in Fig. 3a) associated with a weak embedded low-pressure east of Japan brought a warm, moist air mass northward into the same region. The juxtaposition of these two air masses created a strong temperature gradient, supporting the formation of a strong (>90 ms^−1^ at the 250 hPa level) west-east oriented jet over the western Pacific (Fig. 3a), which propagated eastward into the central Pacific (Fig. 3b). Another weak low developed along the frontal zone east of Japan from 22 to 24 June (Fig. 3c, low centered at 39 N, 157E). Southerly flow ahead of lows and frontal zones, as well as diabatic (condensational) heating within clouds in frontal zones typically contribute to low-level warming downstream of such features, known to be associated with building high-pressure ridges. By 24 June, ridging had developed within the jet downstream of both lows (Fig. 3c).

From 23 to 24 June the upper-level wave (from Fig. 3b) transited the Pacific, deepening the central Pacific upper-level low (or trough) and maintaining a long frontal zone extending equatorward of this low (Fig. 3c). South-southwesterly flow ahead of and within this frontal zone, and diabatic heating within it, brought warmer air into the eastern Pacific, building high pressure ridging over the eastern Pacific (Fig. 3c)—the high-amplitude ridge associated with this unprecedented heatwave. This frontal zone persisted (Fig. 3d), gradually weakening into 28 June, while continuing to supply diabatic heating and southerly flow to maintain the downstream ridge. Quasi-stationary high-pressure ridges such as this are also known as blocking highs or atmospheric blocking.

**Fig. 4:**
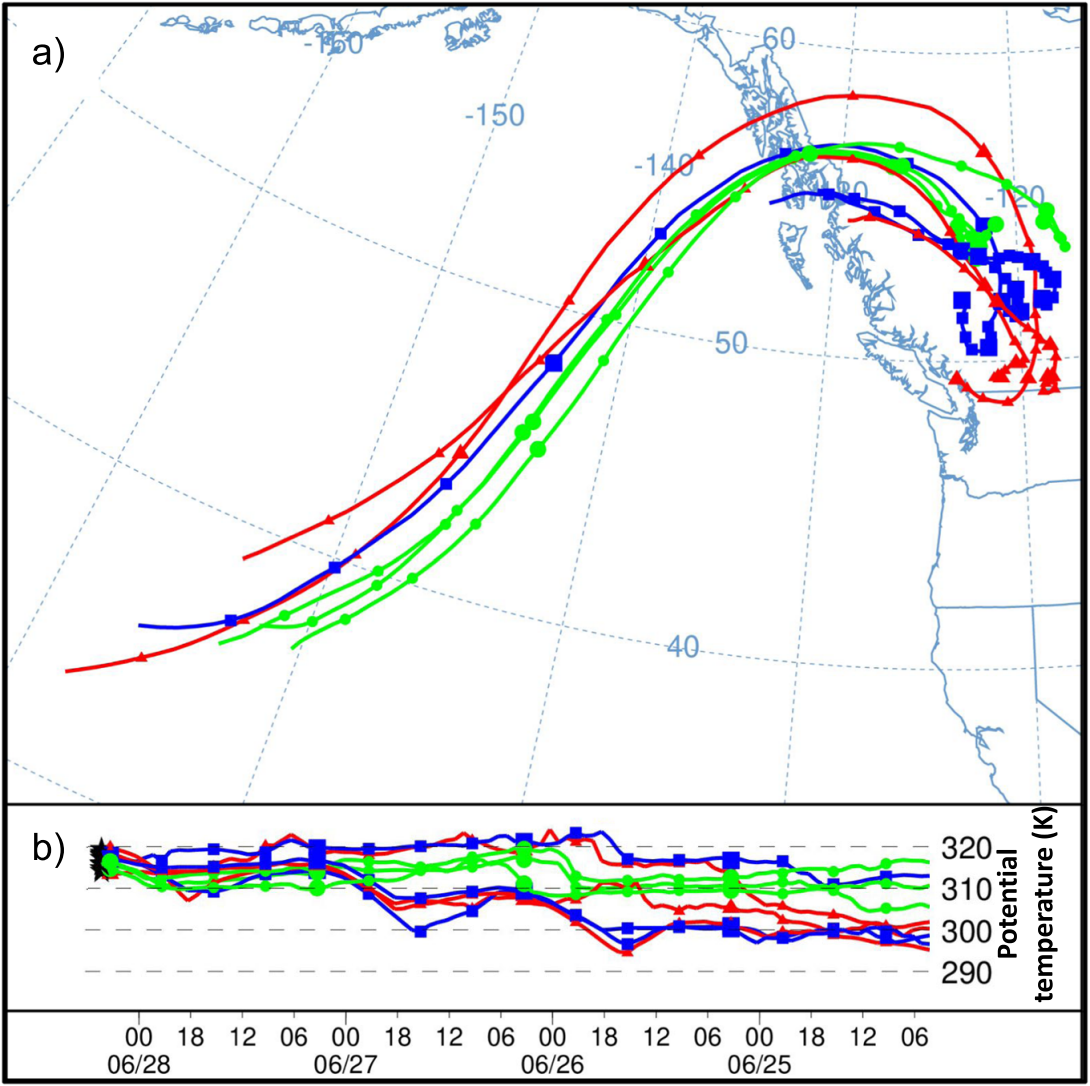
Back-trajectories for Near-surface Heatwave Air. **a** Nine representative four-day backward trajectories terminating at 500 m above ground level (AGL) within the strongest heat anomaly (boxed area in Fig. 1a). **b** Potential temperature of parcels along the trajectory, time from right to left. Potential temperature changes indicate diabatic heating/cooling. Small (large) markers indicate every 6 (24) h, and colors correspond to the trajectory terminating latitude. Trajectories were computed and plotted using GFS 0.25° forecast data and the NOAA HYSPLIT model.

Four-day backwards air parcel trajectories from GFS forecast data produced using the NOAA HYSPLIT model^5^ (Fig. 4 and Supplementary Fig. S1) reveal that the low-level air mass over the PNW heatwave region warmed because of: (1) upstream diabatic heating within frontal zones over the eastern Pacific on 24 and early on 25 June, and within orographic clouds over the Alaska Panhandle late on 25 June (presumably predominantly condensational heating), (2) adiabatic warming as it subsided under strong high-pressure ridging, and (3) diabatic heating over the land region under clear sky conditions (presumably predominantly shortwave radiative forcing^6^ and entrainment/mixing); the heatwave occurred notably close to the summer solstice when insolation is maximum in the Northern hemisphere. Analysis of 63 trajectories shows that ~78% (~14 K) of the net temperature change of the trajectory parcels over the 4 days resulted from diabatic processes, while ~22% (~4 K) was due to adiabatic processes associated with net subsidence (see Methods). This analysis uses forecast data, not observations or reanalysis; however, in the next section, we show that near-term forecasts of this event were good, albeit underestimating the magnitude of the maximum temperatures.

Other studies also provide evidence that sensible heating, mixing, and subsidence played significant roles in locally heating the low-level air mass of this heatwave^7–9^. Neal et al.^8^ emphasized the influence of diabatic heating on the formation of the blocking high pattern, but did not quantify this effect—our trajectories provide a quantitative estimate of the importance of this role, and corroborate their findings on diabatic and adiabatic processes. Soil moisture feedbacks likely also played a role in the high temperatures of this unprecedented heatwave^9,10^.

Lytton, which recorded the heatwave’s hottest temperature, lies within an arid, steep, rocky canyon. The aridity means that a relatively small portion of incoming solar radiation goes into latent heating, leading to a relatively larger magnitude of sensible heating. This heating was compounded over multiple days; factors contributing to limited overnight cooling may have included (1) the thermal inertia of the rocky terrain, and (2) the steep canyon walls creating a smaller sky view factor, limiting longwave radiation loss to space. For coastal population centers (e.g., Vancouver, BC; Seattle, Washington; Portland, Oregon), a coastal thermal trough^11^ enhanced the coastal pressure gradient, bringing so-called “outflow” winds from the interior to the coast. These may have provided additional warming because (1) hotter air from the interior was transported to the coast, and (2) adiabatic warming occurred as the air traveled from higher interior elevations towards the lower-elevation coastal regions.

This event was widely described in the media as a “heat dome”, wherein subsidence/adiabatic warming, “trapped air”, and sensible heating are the dominant mechanisms driving the anomalous heat. This conceptual model ignores the role of upstream diabatic heating, which our analysis shows is a significant heat source. This “heat dome” phrase is not a common phrase within the scientific community; a search indicates the phrase has only been used by nine papers in the American Meteorological Society database (and most of these refer to “urban heat domes,” not a synoptic scale feature). We, therefore, refer to this event as a “blocking high”, “heatwave”, or “heat emergency”.

### Forecasts

We consider the evolution of an operational weather forecast for this event during the 8 days prior to the heatwave onset on 25 June (heatwave timing is shown in Fig. 1b), and provide an operational meteorologist’s perspective with respect to the interpretation and communication of the forecasts. We focus on forecast data from the 42-member North American Ensemble Forecast System (NAEFS)^12^, which is a combination of the Canadian Ensemble Forecast System (CEFS)^13^ and U.S. Global Ensemble Forecasting System^14,15^. We also include a comparison of deterministic forecasts from the Canadian Global Deterministic Prediction System (GDPS)^16^ and the U.S. Global Forecast System (GFS) v16^17^. The NAEFS forecasts presented here underwent bias correction and probabilistic calibration (similar to the methods described in Bourdin et al.^18^) as part of an operational forecast. These data are presented as an example of the type of information and skill forecasters can obtain from a typical modern medium-range operational forecast system for such an extreme event as this, but it is not our intent to provide a full evaluation of this forecast system; such an evaluation is provided for the ECMWF forecast by Emerton et al.^19^. More details on ensemble forecasting are provided in the Methods section.

On 18 June, 8 days before heatwave onset on 25 June (forecast terminology counts 18 June as day 1) and 12 days before temperatures peaked on 29 June (see Fig. 1b), the ensemble forecast gave its first indication of a heatwave. Long-lead-time ensemble-average forecasts of extreme events are moderated by the divergent scenarios of the ensemble members (however, lower-probability more-extreme scenarios are also considered). Given the known lower forecast skill at longer horizons, and the moderate heatwave forecast by the ensemble-average, meteorologists communicated that a heatwave was likely for the following weekend.

By 21 June, 5 days before onset and 9 days before peak temperatures, the forecast ensemble clearly indicated an extreme heatwave, with high confidence that temperatures would exceed the 95^th^ percentile of the climatological distribution for several days (see Supplementary Fig. S2). From experience forecasters knew this meant daily records would likely be broken (hottest temperature recorded for a given calendar day), and all-time records (hottest temperature recorded on any day) looked possible. Deterministic forecasts from 21 June also indicated extreme temperatures (e.g., Supplementary Fig. S2); however, forecasters are less likely to trust such single model forecasts at long forecast horizons, especially when they indicate extreme events, as they are more prone to false alarms. Meteorologists are typically reluctant to make extreme weather forecasts at forecast horizons of around a week for fear of “crying wolf” and the associated reduction in end-user trust. In this case, however, the ensemble forecast provided sufficient certainty that meteorologists were able to warn of “extreme” heat at this relatively long-lead time—a testament to ensemble forecast technology.

From 23 to 25 June, the forecasted “most likely” temperatures continued to increase, as well as the longevity of the heatwave, particularly for interior BC (see lower panel in Supplementary Fig. S2, showing forecasts initialized on 24 June). Meteorologists communicated certainty about all-time records being broken, and warned about potential impacts, demonstrating that the forecasts were able to capture the unprecedented nature of this event. By the onset of the heatwave on 25 June (5 days before it peaked), forecasts of the magnitude and longevity of the event were very good; although forecasted high-temperature records fell 1–3 °C short of the observed highs in many cases.

### Subseasonal forecasts

The ability to forecast extreme events at longer lead times can significantly enhance early warning and preparation systems. Subseasonal to seasonal (S2S) forecasts fill the gap between the short to medium-range weather forecasts discussed in the previous section, and seasonal forecasts (3 months lead time and beyond). We analyze the prediction of this heatwave event using the European Centre for Medium-Range Weather Forecasts (ECMWF) operational S2S forecasts. We look for prediction of temperatures or frequency of atmospheric blocking above the 95th percentile (referred to as ‘extreme’ in the following text), as no ensemble members of any S2S forecasts predicted temperatures as extreme as those observed for initializations earlier than 17 June^20^.

**Fig. 5:**
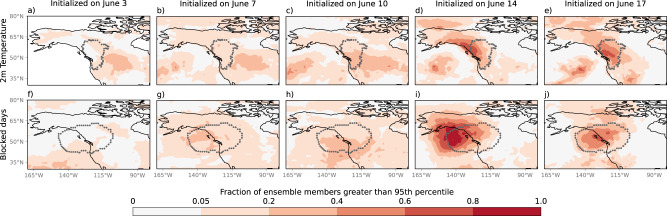
Subseasonal forecasts of extreme temperature and atmospheric blocking. Fraction of ensemble members that predicted 2 m temperature (**a**–**e**) and number of blocked days (**f**–**j**) greater than the 95th percentile of the respective climatologies during 25 June to 1 July. For a 95th percentile event, 0.05 is the statistically expected value if there is no forecasting skill. Forecasts were initialized on 3 June (**a**, **f**), 7 June (**b**, **g**), 10 June (**c**, **h**), 14 June (**d**, **i**), and 17 June (**e**, **j**). Gray contours show the observed location of the heatwave based on ERA5 2 m temperature data.

Figure 5 demonstrates that forecasts initialized from 10 June onwards showed an increased probability of extreme temperatures from 25 June–1 July, a lead time consistent with earlier studies for this^20^ and other^21^ heatwaves. From 14 June, up to 70% of ensemble members forecasted extreme temperatures somewhere within the affected region, with the predicted location improving with shorter lead times. An increased probability of extreme blocking was seen in forecasts at lead times as early as 7 June. This is not associated with a large increase in the probability of extreme temperatures over the affected region, likely due to errors in forecasted block location placing the block too far west over the ocean. For forecasts initialized on 3–10 June, a higher probability of extreme temperature anomalies was predicted to the east of the heatwave region, consistent with a widespread soil moisture deficit across this region at the time (see Supplementary Figure S3).

Interestingly, a stronger forecast in terms of both temperature and blocking frequency was seen for forecasts initialized on 14 June relative to forecasts initialized on 17 June (Fig. 5d, e, i, j). Similar behavior has been seen in other heatwave forecasts at longer lead times^21^. The location of the extreme temperature anomalies and blocking did, however, improve with decreasing lead time, with the 14 June forecasts typically placing the block too far to the west over the ocean (Fig. 5i).

### Climate change context

“What was the role of anthropogenic climate change in this event?” is one of the first questions asked by the public and policy-makers alike. Despite recent progress in extreme event attribution science^22^, this is a difficult question to answer for extremely rare events. One common approach is to quantify the change in the return period of an event; however, the validity of this method is uncertain for events this rare^23–25^. Of the papers published on this event thus far, estimates of the current return period of this event vary from 1 in 10,000–100,000 years^24^, 1 in 1000 years^25^, to 1 in 200 years;^10^ further research on methods to estimate the expected occurrence of such unprecedented extremes in the context of a limited observational record and a changing climate are critically needed.

It is clear, however, that anthropogenic warming of the planet contributed to the severity of this event^23,25,26^—mean global temperatures for 2010–2019 were around 1.1 °C warmer than in 1850–1900, with the Intergovernmental Panel of Climate Change (IPCC) attributing this increase entirely to human activities^27^. Land temperatures have warmed faster than the global mean (1.5 °C since 1850–1900), and the rate of warming has increased over the past 70 years^27^, increasing the probability of record-shattering extremes^28^. Regionally, this PNW heatwave occurred in a background summer climate that was ~1.0 °C warmer than at the end of the 19th century (see Methods), although changes in extremes do not necessarily mirror changes in the mean temperature^29^.

This heatwave broke many records by substantially more than the observed change in mean summer temperature of 1.0 °C (see Fig. 2). Whilst this is at least partially due to internal (i.e. unforced) variability of the climate system, the question remains whether there was a dynamical impact of climate change in addition to the thermodynamic impact of increasing mean temperatures. The concerning possibility is that anthropogenic climate change may have made the atmospheric circulation patterns that led to this event stronger or more likely. This is an area of ongoing research, with many possible mechanisms to be explored and understood^30^ and we are still far from a full understanding and community consensus.

One proposed hypothesis that has garnered a lot of attention, including in the media, is that Arctic amplification (AA), by reducing the equator-to-pole temperature gradient, reduces the strength of the mid-latitude atmospheric jetstream, which may lead to amplified mid-latitude atmospheric waves associated with heatwaves^31^. There is still substantial disagreement in the scientific community regarding this hypothesis however^32,33^. One recent study suggests that regional sea-ice loss may contribute to increased heatwave frequency in this Western North America region^34^. To investigate if surface AA-induced changes to atmospheric dynamics may have increased the probability of this heatwave, we perform analysis using two metrics of atmospheric waviness, including a proxy for atmospheric blocking (see Methods) on two simulations from the Polar Amplification Model Intercomparison Project (PAMIP)^35^. The PAMIP simulations have been shown to simulate a weakening of the high-latitude jet in response to AA;^36^ however, we find no evidence for robust changes in waviness over North America in response to sea-ice loss or sea surface temperature changes in the two PAMIP models (those with available daily data; see Methods). The lack of changes seen in our analysis should not be taken as conclusive evidence that AA did not contribute dynamically to this event. A more complete analysis of the role of AA, including using additional metrics associated with heat extremes and investigation into the role of the depth or localization of the AA, is required to fully answer this question.

Other potential mechanisms for how climate change may have influenced this event through impacts on atmospheric circulation include changes in: soil moisture affecting extratropical Rossby wave sources^37^ and land-atmosphere feedbacks on the development of atmospheric blocks^10,38,39^; tropical Rossby wave sources from changing tropical convection^40^; and Rossby wave propagation^30^, including changes in atmospheric waveguide conditions that may lead to enhanced amplification of stationary waves^41^. In the Synoptic Conditions section, we highlight the important role of diabatic heating within upstream frontal zones for the development of the atmospheric block critical to this heatwave, consistent with other recent studies on atmospheric blocking^42^. Given increases in atmospheric water vapor content in a warmer world^43,44^, this impact of climate change may influence the strength and development of blocking highs, and the near-surface temperature anomalies within them. Mo et al. (2022)^45^ suggest that water vapor and sensible heat transported within an upstream atmospheric river prior to this heatwave may have played a role in the extreme temperatures, adding another mechanism through which climate change may impact heatwaves. With so many potential mechanisms, understanding the role of climate change on the atmospheric dynamics associated with heatwaves is understandably difficult; however, the impacts of this unprecedented event, summarized in the remainder of this paper, illustrate the importance of this research.

### Impacts

The impacts of this unprecedented heatwave were felt across many spheres of our ecosystem. Whilst the extremely high temperatures lasted for around a week (Fig. 1b), some impacts continued long after. We provide an analysis of a number of these impacts, in chronological order. We start with those felt mostly during the heatwave, including human health impacts and coastal marine die-offs. Next, we consider impacts that were initiated during the heatwave but continued to be felt for weeks to months after, including wildfires, agricultural yield declines, and glacier/snow melt. Finally, we end with the landslides that occurred throughout the summer and fall of 2021, influenced by cascading effects initiated by the heatwave.

### Human health

Heatwaves can have catastrophic impacts on human health. Substantial increases in death rates have been documented during and immediately following past heatwaves, such as those occurring in Chicago in 1995^46^, western Europe in 2003^47^, and Moscow in 2010^3^. During this 2021 PNW heatwave, increased mortality was reported in the Canadian provinces of British Columbia and Alberta and the US states of Washington, and Oregon. Here we report data from the most recent reports, from governmental sources where available (see Methods for more details on data sources and reporting). Whilst estimates of the total number of deaths due to this heatwave are likely to change over time as the event receives further study, the evidence suggests that the death toll has little precedent in the affected regions, particularly BC.

Between 25 June and 2 July, an estimated 740 excess deaths in the province of BC were observed^48^, a 95% increase in population mortality over an 8-day period. Thus far, the BC Coroners Service has attributed 619 deaths to the extreme heatwave event, with most (93%) of these deaths occurring between 25 June and 1 July^49^. In other regions, reports indicate at least 66 attributable deaths in Alberta^50^, 100 heat-related deaths in Washington State^51^, and 83 heat-related deaths in Oregon State^52^, giving a total preliminary estimate of at least 868 deaths across the PNW that have been attributed to this heatwave.

Most deaths during the heatwave occurred in private residences^49,51^, likely due to dangerously high indoor temperatures^53^. Initial analyses of the community deaths in metropolitan Vancouver (the largest city in BC) found that they were disproportionately in neighborhoods with higher material and social deprivation and lower levels of green space^53^, with higher risk among those aged 65–84 years and among females. Different analyses from the BC Coroners Service also document that severe mental illness and substance use disorder were significant risk factors^49^. Further analyses are needed to account for both of these interrelated factors.

Serious but non-fatal health impacts also occurred. From 25 to 30 June the average daily number of emergency department visits for heat-related illnesses in the US Health and Human Services Region 10 (which includes the states of Washington, Oregon, Idaho, and Alaska) was 69 times higher in 2021 than during the equivalent days in 2019, with disproportionate impacts on males and persons aged 75 and older^54^. In Canada, WorkSafe BC, an agency dedicated to promoting safe and healthy workplaces across the BC province, advised employers to consider workplace closures during the extreme heat event if they did not have air conditioning, and numerous businesses, particularly restaurants, followed this advice.

From a public health perspective, it is important to reflect on the fact that the heatwave occurred after 18 months of a global pandemic. Social isolation is a known risk factor for heat-related deaths^55,56^, and there is ample evidence that social isolation increased drastically during the pandemic, especially among older adults^57^. As such, the COVID-19 pandemic response likely primed the most at-risk population to be particularly susceptible to this extreme heat event. Public use of interventions such as cooling centers may have been reduced due to perceived COVID-19 risk, in addition to the pandemic response and concerns around COVID-19 creating issues with staffing such interventions^49^.

### Marine life

**Fig. 6:**
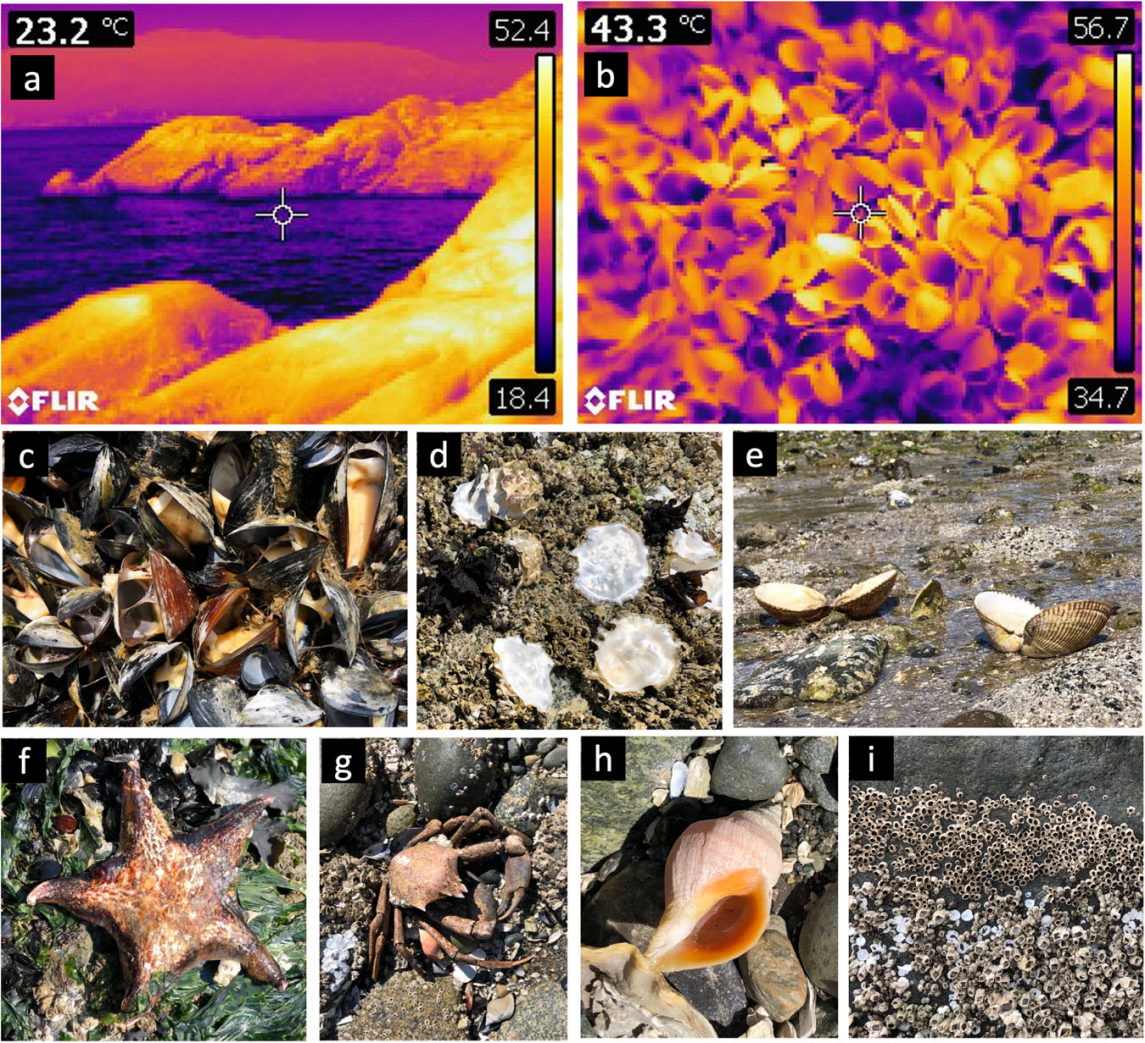
Heatwave impacts on marine life. Thermal images showing extreme high surface temperatures during low tide on 28 June, 2021, on **a** a rocky intertidal shoreline and **b** within a mussel bed. Scale bars indicate the range in temperature from the coolest to warmest parts of the image, while the value at the upper left indicates the temperature in the cross-hairs at center. Note that the mussels in **b** have died and are gaping open. A subset of species impacted by the heatwave are shown in **c**–**i**, including **c** bay mussels, *Mytilus trossulus*, **d** Pacific oysters, *Magallana (= Crassostrea) gigas*, **e** heart cockles, *Clinocardium nuttallii*, **f** leather stars, *Dermasterias imbricata*, **g** kelp crabs, *Pugettia producta*, **h** dogwhelks, *Nucella lamellosa*, and **i** barnacles, *Chthamalus dalli* (upper portion of image) and *Balanus glandula* (lower portion of image). See Methods for locations and dates of photos in **c**–**i**.

Rocky intertidal shores are some of the most physically stressful habitats on Earth, and many of the species that occupy them often live very close to their physiological tolerance limits^58^. Intertidal ecosystems are therefore often used as bellwethers for the ecological effects of climate change and extreme weather events^59^. Plants and animals that live in the intertidal zone are especially susceptible to extremely high temperatures during daytime low tides, when solar radiation can raise organismal body temperatures well above air temperature^59,60^. The hottest days of the 2021 heatwave coincided with very low, early afternoon low tides throughout most of the Salish Sea (the inland waters of BC and Washington State). As a result, surface temperatures in excess of 50 °C were observed in the intertidal zone (Fig. 6a, b), particularly on gently sloping south and west-facing surfaces that received the most direct solar radiation.

As the lethal thermal limits for even the most tolerant local intertidal species are well below 50 °C, there was extensive mortality for numerous species at low tide. Species affected included barnacles, mussels, oysters, clams, gastropods, crabs, sea stars, and more (Fig. 6). Sessile species, which are unable to retreat to cooler microhabitats, were especially adversely impacted. Barnacle and mussel mortality, and the accompanying foul odor, was reported by researchers and concerned citizens from the southern end of Puget Sound, Washington State, to the BC Central Coast^61^. Mortality was particularly pronounced in the Strait of Georgia, where temperatures were especially high, and low tides were centered in the middle of the afternoon.

On one representative mussel-dominated shoreline, the bay mussel (*Mytilus trossulus*) mortality rate was in excess of 70%, and over one million mussels were estimated to have died in a mere 100-m stretch of shoreline (see Methods). Surveys on a barnacle-dominated shoreline documented barnacle mortality rates that were also in excess of 70% even after factoring in pre-heatwave background mortality; at this site, the heatwave killed ~10 million barnacles (*Balanus glandula*) along a single 100-m stretch of intertidal habitat (see Methods). While precise estimates for total heatwave-induced mortality along the highly heterogeneous ~7500 km coastline of the Salish Sea are difficult to make, similar magnitudes of proportional barnacle and mussel mortality were widely observed, and the total number of marine invertebrates killed was almost certainly in the billions.

### Wildfires

**Fig. 7:**
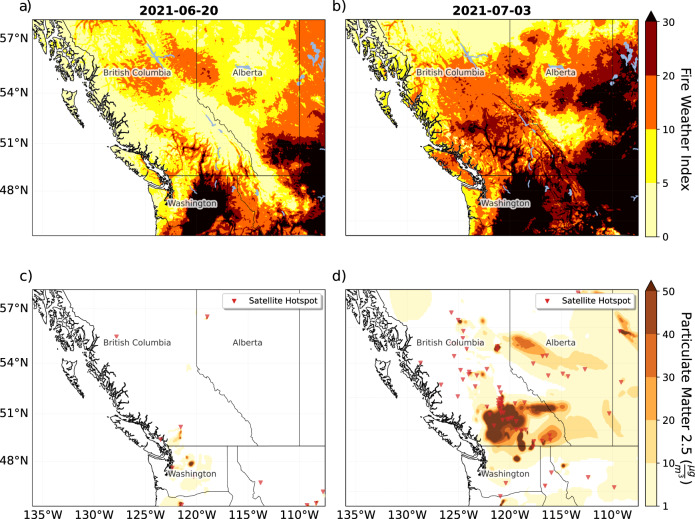
Heatwave impacts on wildfire conditions. Model estimated Fire Weather Index (FWI; **a**, **b**) and smoke concentration and satellite hotspots (**c**, **d**) for pre-heatwave conditions on 20 June (**a**, **c**) and post-heatwave conditions on 3 July (**b**, **d**). Smoke concentration in **c**, **d** is represented by the concentration of particulate matter (PM) 2.5. Satellite hotspots in **c**, **d**, indicating likely wildfire activity, are shown by the red triangular markers.

The persistent hot and dry conditions associated with the heatwave cured forest vegetation, resulting in extreme fire danger and increased wildfire activity. Figure 7 shows the increase in Fire Weather Index (FWI; see Methods), modeled smoke concentration, and satellite-detected hotspots between 20 June (pre-heatwave) and 3 July (post-heatwave). Over this period, BC went from six active wildfires with 123.5 hectares burned to 175 wildfires consuming 78,939 hectares. On 30 June, observed FWI values in Lytton, BC, reached an extraordinary value of 132 (typical values are between 0 and 30). By 11 July, the Canadian Interagency Forest Fire Centre (CIFFC) increased Canada’s National preparedness level to 5, its highest rank: wildfire suppression resources were limited, and aid from international agencies was requested.

During the heatwave itself, two noteworthy fires were discovered actively burning within the BC Southern Interior: the Sparks Lake Fire (discovered 28 June), and the McKay Creek Fire (discovered 29 June). Both are suspected to be human-caused, and were located in the Southwest Interior of BC, a zone that experienced some of the hottest and driest conditions throughout the heatwave. On the afternoon of 29 June, mid-tropospheric moisture moved into the high-pressure atmospheric ridge over the heatwave region, helping produce the thermodynamics for pyrocumulonimbus flammagenitus (CbFg) clouds to form over these burning wildfires. These CbFg clouds can produce large amounts of lightning, providing ignition for more fires^62,63^. Combustion of the forest vegetation produces smoke particles and releases additional water vapor into strong thermal updrafts above wildfires^62^. The combined smoke and water vapor rise to a cloud base, where they cool and the water vapor condenses into cloud droplets; this led to the development of CbFgs over each wildfire in the late afternoon on 29 and 30 June. On 30 June, the CbFgs were highly convective, producing positively charged anvil cloud tops that propagated northward (as determined from GOES-17 visible satellite imagery). Over the evening hours of 30 June approximately 120,800 cloud-to-ground lightning strikes occurred^64^, with the CIFFC reporting at least 127 new lightning-ignited wildfires from 30 June to 2 July.

The high-pressure atmospheric ridge began to breakdown ~30 June. This led to hot, dry, and now windy and unstable conditions, which fanned the flames of wildfires ignited by the CbFg lightning and enabled the rapid growth of these fires. The continued breakdown of the ridge and a cold frontal passage yielded more CbFgs and lightning with little precipitation. This cycle occurred each afternoon during the first week of July and ignited an average of more than 40 new wildfires each day in BC.

### Agricultural yields

The heatwave had pronounced impacts on agriculture in the Canadian provinces of British Columbia and Alberta, notably occurring during crucial growing stages of many crops. BC has the highest diversity of crops in Canada, with major field crops of canola, oats, wheat (particularly spring wheat), and barley, and produces more fruit than any other Canadian province^65^. Extreme weather events such as heatwaves can severely impact annual yields^66^, with temperatures exceeding a crop-dependent threshold resulting in substantial yield drops^67^. Yields in many crops show robust trends over time (often positive) due to a combination of technological and climate changes. Using estimated field, vegetable, and fruit yields from Statistics Canada (see Methods), we calculate linear regressions to estimate historical trends for each crop and to produce a predicted 2021 yield. We also calculate the standard deviation, *σ*, of de-trended annual yields to quantify interannual variability. We compare the estimated 2021 yields to our predicted yields and interannual variability: of the 26 field, fruit, and vegetable crops for which data are available, 24 showed decreases relative to the predicted yield in 2021 (see Supplementary Fig. S4). It is, however, extremely difficult to isolate the impacts of one particular event on annual yield data^66^; we thus also use weekly resolution, satellite-derived Normalized Difference Vegetation Index (NDVI; see Methods) to analyze the timing of changes in crop greenness relative to the heatwave. Reductions in NDVI indicate reductions in plant greenness, and therefore plant tissue damage, reduced growth, and/or reduced plant density.

In British Columbia, estimated field crop yields are substantially lower than predicted for many crops, with spring wheat reduced by 31% (1.9σ), barley by 30% (2σ), and canola by 21% (1.6σ). Oat yields saw smaller declines with 2021 yields only 5% (0.4σ) below-predicted yields; this smaller decline is consistent with 95% of the oat crop being grown in Peace River which experienced slightly less extreme temperatures during the heatwave than other regions (see ‘PR’ crop region in Fig. 1a and in Supplementary Fig. S5, which shows absolute maximum heatwave temperatures), although varying irrigation practices in different regions and for different crops may also play a role. Similar yield declines were seen in Alberta, with many field crop yields the lowest since the widespread drought of 2002, including canola (32% lower than predicted values), spring wheat (31%), barley (32%), and oats (29%). Anomalies for field crop yields in Alberta were all greater than 3σ (see Supplementary Fig. S4).

The production of many major fruit crops also faced major declines in 2021; BC’s soft fruit production occurs predominantly in the Thompson-Okanagan (TO) region, which saw particularly extreme heatwave temperatures (see ‘TO’ in Fig. 1a and Supplementary Fig. S5). Decreases relative to predicted yields were greater than 2σ for sweet cherries, grapes, plums, and raspberries, with declines greater than 1σ in apples, nectarines, peaches, and pears. Not all fruit crops suffered equally, with, for example, cranberry yields only 2% below predicted, and in fact 10% above the 2011–2020 average due to a strongly positive historic trend. Several factors may influence these differences, including: timing of critical growth stages relative to the heatwave (e.g., sweet cherries and raspberries are harvested in July/August, and are thus likely to be in a critical growth stage in late June, whilst cranberries are harvested later); irrigation practices (cranberries have high irrigation coverage relative to many other crops); as well as spatial differences in the local temperatures. Vegetable yields also suffered in 2021 for many crops, with pumpkins, tomatoes, and radishes declining by greater than 2σ, and Brussel sprouts, lettuce, green peas, squash, and zucchini yields reduced by greater than 1σ (see Supplementary Fig. S4). The livestock sector in BC also experienced significant mortalities during the heatwave, with reportedly at least 651,000 farm animals dying between 24 June to 30 June^1^.

**Fig. 8:**
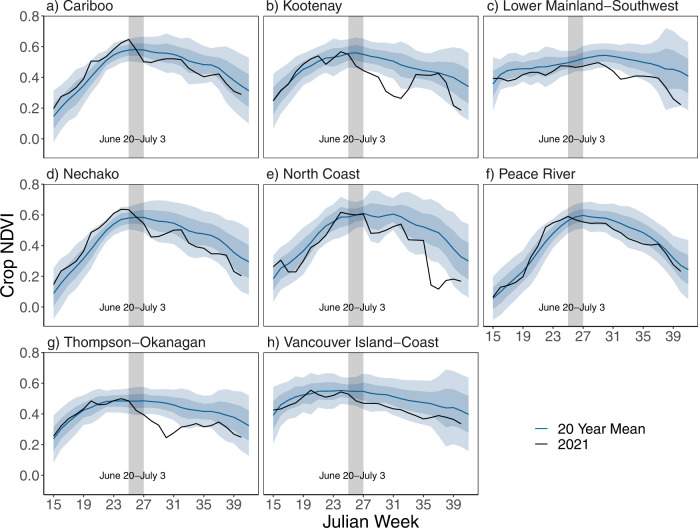
Heatwave impacts on crop health. Weekly Normalized Difference Vegetation Index (NDVI) from the Crop Condition Assessment Program (see Methods), spatially averaged over agricultural divisions in British Columbia, organized alphabetically. 2021 values are shown in the black line, with the 2000–2020 climatology in blue and shading showing interannual variability for this 2000–2020 period (±1 and 2 standard deviations). The gray highlighted region shows the heatwave period, 20 June—3 July; data are reported as weekly averages and so the heatwave is split between weeks 20–26 June and 27 June—3 July. The locations of the 8 agricultural divisions are shown in Fig. 1a in gray contours.

Weekly averaged NDVI values for cropland in each of BC’s agricultural regions are shown in Fig. 8, allowing analysis of the timing of changes in crop greenness relative to the heatwave period. In six out of the eight BC agricultural divisions, a noticeable drop in NDVI occurred during the heatwave period, with the strongest within-heatwave declines in the Cariboo, Kootenay, and Thompson-Okanagan regions, which saw some of the highest temperatures during the heatwave (see crop regions C, K, and TO in Fig. 1a and Supplementary Fig. S5). The timing of these decreases suggests that the unprecedented heatwave likely played a significant role in the annual yield declines recorded. Regional differences in NDVI decreases are likely a combination of differences in local meteorological conditions during the heatwave, dominant crops and their heat sensitivity/tolerance, and irrigation practices.

### Glacier and snow melt

The unprecedented heatwave had a pronounced influence on the region’s cryosphere and hydrology both during the event and for months after. The heatwave-induced streamflow response was spatially variable across BC, in large part due to the variation in snowpack and glacier coverage throughout the province (see Supplementary Fig. S6a).

**Fig. 9:**
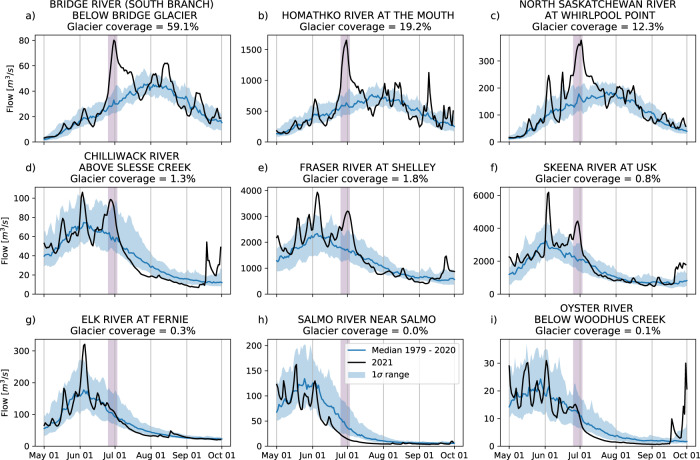
Heatwave impacts on streamflow. Streamflow observations at nine stream gauge stations in 2021 (black line) relative to the 1979–2020 median (blue line) and 1 standard deviation range (shaded). Gauges are organized from top to bottom by basin glacier coverage: highly glaciated basins (**a**–**c**), lightly glaciated basins (**d**–**f**), and minimally or non-glaciated basins (**g**–**i**). See Supplementary Fig. S6 for locations of these gauges.

The exceptionally warm temperatures and clear sky conditions drove rapid ice and snow melt, leading to substantially increased streamflow in basins that had snow or ice available to melt (Fig. 9a–f). In many cases, daily record high flows were achieved, with some all-time records broken (Supplementary Fig. S7). The rapid increase in streamflow led to flood warnings for several downstream communities and an evacuation order in the Pemberton Valley. In some cases, melt rates and daily streamflow rates decreased during the heatwave due to snowpack depletion, despite continuing extreme temperatures (e.g., Fig. 9d, f). Rainfall on 1–2 July in the Rocky Mountains near the BC/Alberta border exacerbated flooding and damages^68^, leading to substantial damages within BC’s Mount Robson Provincial Park, and the helicopter evacuation of hikers^69^.

Glacier melt is an important control of interannual summer streamflow variability^70,71^. The extensive snow melt during the heatwave exposed darker, lower-albedo glacier ice. Combined with persistent warm conditions through much of July this resulted in large amounts of seasonal glacier melt. While basins without substantial glacier coverage experienced lower than normal flows, the influx of glacier meltwater in glaciated basins resulted in late summer streamflows similar to the historical average (contrast Fig. 9a–c with 9g–i; see also Supplementary Fig. S6b), despite the substantial loss of snowpack during the heatwave. The ability of glaciers to sustain normal flows is notable considering the unprecedented nature of the heatwave, and came at the cost of substantial glacier mass loss^72^. The ability of glaciers to compensate for such extreme events is expected to diminish in the future with continued climate change^73^.

### Landslides

Wildfires can result in an increased risk of flooding, erosion, and landslides, due to impacts on both vegetation and soil^74^. Such impacts include a reduction of infiltration rate and water storage capacity, an increase in the susceptibility of the soil to erosion by raindrops, and in some cases increased water repellency of the soil below the surface^74,75^. These changes increase the susceptibility of slopes to debris flows, with studies showing that post-wildfire debris flows often occur in areas with moderate to high burn severity^74,76^. Extreme temperatures can also open desiccation cracks in fine-grained (clay and silt-rich) sediments. Those cracks can later allow ingress of rainwater to potential failure planes at depth which may exacerbate landslide hazards well after the heatwaves and wildfires have ended. Processes associated with wildfires in BC during 2021 are twofold. Firstly, the loss of vegetation and hydrophobicity of the soils led to a more pronounced stream runoff response with steeper hydrographs. Secondly, the added sediment charged the receiving rivers with excess sediment, locally aggrading channel beds and encouraging avulsions and bank erosion.

Hundreds of post-wildfire debris flows were triggered by rainstorms in the summer and fall of 2021 in southwestern BC (e.g., Supplementary Fig. S8). One of the largest wildfires associated with this heatwave was the Lytton Creek Fire, which began south of the village of Lytton and burned ~84,000 hectares^77^. In addition to the extensive damages to Lytton and Lytton First Nation reserves previously described, post-wildfire debris flows affected railway and highway infrastructure. On 16 August, debris flows impacted two semi-trailers and a car on the TransCanada Highway, with no fatalities reported. Further post-wildfire debris flows that damaged infrastructure but did not impact vehicles occurred on 17 September, and during an extreme atmospheric river event on 14–15 November.

**Fig. 10:**
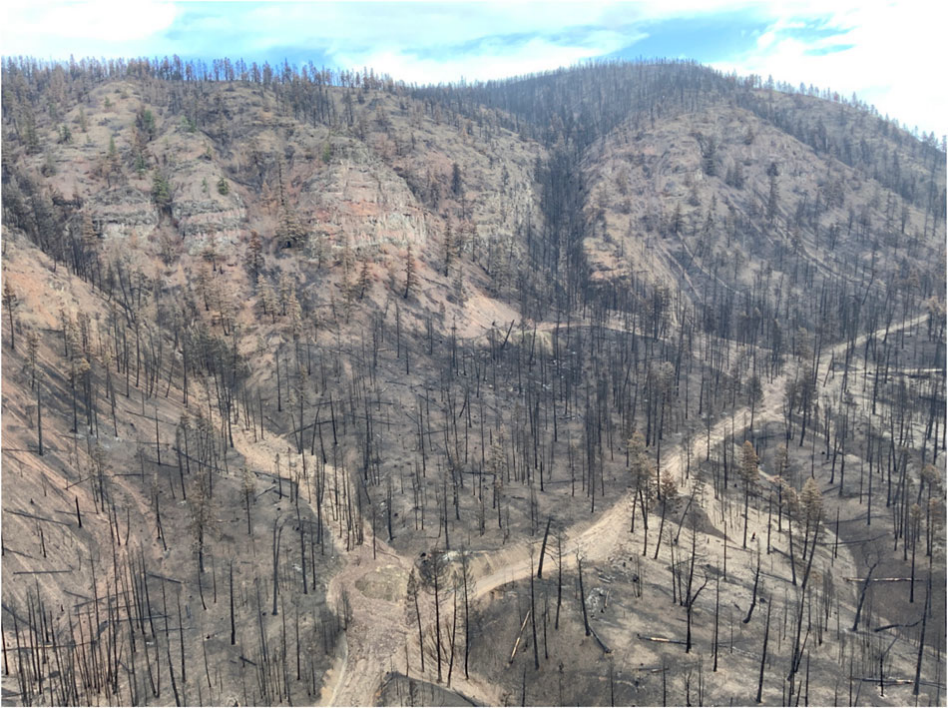
Example of post-wildfire debris flows following the heatwave. Post-wildfire debris flows in Nicoamen River watershed triggered by a rainstorm on 16 August 2021. Severely burnt vegetation is shown by blackened trees. Debris flows originated as slope wash and rilling on steep burnt slopes.

Debris flows following the 16 August rainstorm in the Nicoamen River watershed are shown in Fig. 10. Rainfall rates were not unusually heavy: 4–10 mm h^−1^ over ~4 h, corresponding to a <2-year return period event in this region. The fire-related water repellency in the soil caused soil rilling (a form of erosion) and in-channel sediment mobilization, resulting in abundant sediment transport. Further rainfall in the Nicoamen River watershed during the atmospheric river on 14–15 November remobilized the debris flow sediment as part of a post-wildfire debris flood that undermined three bridge approaches on the TransCanada Highway and one building foundation, resulting in one bridge collapse and necessitating repairs on another. Outside of the Nicoamen River, post-wildfire debris flows and debris floods in the Lytton Creek Fire perimeter severed railway and highway infrastructure (bridges and embankments) in nine other locations^78^. This event likely resulted in the most expensive natural disaster in Canada’s history, with millions of dollars worth of damage along southwest BC’s highways and railways with bridges, railways, and highway embankments failing.

## Discussion

This unprecedented heatwave was one of the most anomalous regional extreme heat events to occur anywhere on Earth since temperature records began^4^. Diabatic processes (condensational heating, sensible heating, entrainment) both upstream of the high-pressure ridge and within it, played a dominant role in heating the air mass. While accurate forecast information was available in the days leading up to the event, and efforts were made to communicate the severity of the event and reduce heat mortality, hundreds of heat-related deaths still occurred, along with other human and ecological impacts. The unprecedented nature of this event made it difficult to anticipate and mitigate these impacts; understanding these impacts may help communities across the world better prepare for the record-shattering extreme heat events that are predicted to occur with increasing frequency as the climate continues to warm^28^.

The impacts of this heatwave highlight the urgency and necessity of improving both warning systems and heatwave preparedness in order to reduce mortality and other human and ecosystem impacts in future extreme heat events. This includes investing in: more research to better quantify and understand how climate change will likely affect extreme events; more research to understand regional impacts of unprecedented heat and how these impacts can be mitigated; improving our early warning systems through both improved forecasts, including on S2S timescales, and increased collaboration and communication between forecast providers and forecast users; and establishing systems and mitigation procedures that can be initiated in response to an early warning. Indeed, among other changes in response to this event, BC has established a new category of warning called an “Extreme Heat Emergency”, producing alerts analogous to similar systems for wildfires and flooding. The intent is to provide an appropriately coordinated and resourced provincial response when the next extreme heat event occurs. As we move into an unrelentingly warmer world due to anthropogenic global warming, extreme heat events across the globe are becoming increasingly common and more intense^28,79^. The extraordinary nature of this extreme heatwave highlights that all jurisdictions should be working to address the inevitable risk of unprecedented heat; in particular, we urge regions and jurisdictions that have not experienced an extreme heat event in recent decades to act now, before such a disaster occurs.

Many of the negative effects of this heatwave were disproportionately felt by First Nations communities (e.g., the Lytton fire, as well as numerous effects of ecosystem impacts on communities with close relationships with the land, water, and natural resources), the elderly, and people living in neighborhoods with higher material and social deprivation, highlighting the importance of climate justice in the response to climate change^80^. Indeed, health vulnerability to heatwaves is known to be distributed unequally^81^, with higher levels of material and social deprivation a vulnerability factor in previous heatwaves as well^82^. There is thus an urgent need to provide better protection and resources to our most vulnerable populations so that natural disasters do not continue to amplify existing inequalities and systemic racism.

The catastrophic impacts of this unprecedented heatwave underscore the importance of understanding extreme weather events and how their frequency or intensity will change with anthropogenic climate change, and, crucially, how to mitigate their impacts. Raju et al. (2022)^83^ argue that disasters occur when hazards meet vulnerability, and that we can, and should, work to reduce both the hazards (through rapid reduction of greenhouse gas emissions to true net-zero, or net-negative) and the vulnerability (through changing social and political systems). This unprecedented heatwave has provided lessons and examples that we can learn from, and provides us with a vivid depiction of why and how climate change can be so devastating.

## Methods

### Overview/introduction

To analyze heatwave temperatures, we use daily maximum 2 m temperatures (TX) from weather stations, and from the ERA5 reanalysis^84^, a gridded dataset that assimilates observations. A previous version of the ECMWF reanalysis, ERA-interim, performs well relative to pure-observation datasets for temperature extremes^85^ and thus we expect ERA5 to provide an accurate assessment. ERA5 data were downloaded from the C3S datastore^86,87^ on a 1° × 1° spatial grid, and daily maximum temperatures were calculated from hourly maximum temperatures. Station data for Lytton, BC, were obtained from the Meteorological Service of Canada; for dates with more than one active Lytton station, averages across stations were taken prior to the calculation of the climatology.

We calculate TX anomalies relative to daily climatology from 1981–2020, and then calculate a 3-day running mean of these anomalies. Figure 1a shows the maximum value of this running mean over the heatwave period 22 June to 3 July 2021. Using temperature anomalies is a useful method of removing spatial differences in absolute temperatures; however, some impacts, such as those on crops or the cryosphere, are often related to absolute temperature thresholds. We therefore also show the maximum 3-day running mean of absolute TX heatwave values in Supplementary Fig. S5.

To analyze record temperature exceedances (shown in Fig. 2) we calculate the maximum TX (TX_X_) over the heatwave period from 22 June—3 July 2021, and for a historical period from 1 January 1950 through to 1 June 2021. The difference between these is shown in Fig. 2, with the 2003 European heatwave (TX_X_ between 1 July—31 August 2003 relative to TX_X_ between 1 January 1950—1 June 2003), and the 2010 Russian heatwave (TX_X_ between 1 July 2010—31 August 2010 relative to TX_X_ between 1 January 1950—1 June 2010).

In Fig. 2a we include record temperature exceedances for observations (shown in the individual markers) from the Canadian Long-Term Climate Extremes database, created by the Meteorological Service of Canada^88^. For this dataset “virtual” climate stations were created by joining together climate data from nearby stations to make long-term records, allowing for longer records than looking at individual stations. We show data only for virtual stations that go back to at least 1949, with the longest record starting in 1874. These data have not been quality controlled to remove artifacts such as discontinuities and non-climate trends; however, the dataset is updated frequently, allowing us to use it for recent events. We expect the ‘record exceedance’ values from individual stations to be generally slightly lower than for the ERA5 dataset, as many of the records are longer, although the spatial averaging of the ERA5 dataset may counter this effect. The general extent and distribution of the record exceedances are similar (see Fig. 2a), with close agreement over the station of Lytton (shown in the red triangle in Fig. 2a), where the new Canadian daily record maximum temperature was set: the ERA5 gridpoint value has a record exceedance of 5.7 °C, while the Lytton station record was broken by 5.2 °C.

### Synoptic conditions

The description of the synoptic conditions was produced through analysis of the evolution of geopotential height, wind speed, precipitable water, relative humidity, temperature, and sea-level pressure fields, at various levels in the atmosphere.

To gain insight into the processes responsible for the low-level temperature anomaly, 4-day backwards trajectories terminating at 500 *m* above ground level (AGL) within the heatwave region (see box in Fig. 1a) at 0100 UTC 28 June (6 pm 27 June local time) were calculated using the NOAA HYSPLIT model using data from GFS 0.25° forecast data (Fig. 4 and Supplementary Fig. S1). To quantify the roles of diabatic vs adiabatic heating, 63 trajectories are analyzed; in the figures, produced using the online version of HYSPLIT, HYSPLIT-WEB^5,89^, only nine trajectories are presented for readability. Both potential temperature changes (to quantify diabatic heating/cooling) and changes in pressure (to quantify adiabatic heating/cooling) along the trajectories were examined. To determine likely physical processes responsible for changes in potential temperature and pressure, parcel positions at the time/location of the changes were cross-referenced with maps of the fields mentioned in the above paragraph (e.g., increases in potential temperature while the parcel was ascending within a saturated air mass suggest condensational heating was the dominant diabatic process).

Changes in potential temperature (*θ*) are solely due to diabatic processes, so temperature changes due to diabatic processes can be calculated simply as:

1 $$\varDelta {T}_{{diabatic}}={\theta }_{2}-{\theta }_{1}$$

where the subscripts 1 and 2 denote values at the start and end of the backwards trajectories respectively. To calculate adiabatic changes, we use the equation for potential temperature:

2 $$T=\theta {\left(\frac{P}{{P}_{s}}\right)}^{\kappa }$$

where $\kappa=R/{c}_{p}=0.2857$, *P* is pressure, and P_*S*_ is standard pressure, i.e., 1000 hPa. The change in temperature due to adiabatic processes (i.e. with no change in *θ*, such that θ_2_ = θ_1_) is $\varDelta {T}_{{adiabatic}}=\,{T}_{2}({\theta }_{2}={\theta }_{1})-{T}_{1}({\theta }_{1})$, i.e.:

3 $$\varDelta {T}_{adiabatic}={\theta }_{1}\left[{\left(\frac{{P}_{2}}{{P}_{s}}\right)}^{\kappa }-{\left(\frac{{P}_{1}}{{P}_{s}}\right)}^{\kappa }\right]$$

$\varDelta {T}_{{adiabatic}}$ and $\varDelta {T}_{{diabatic}}$ were calculated for each of the 63 trajectories, yielding average values of 14.00 K and 3.98 K, respectively, with a total change of 17.98 K. This gives estimated contributions of 78% and 22% for diabatic and adiabatic heating respectively. The relative contributions of diabatic and adiabatic processes can also be seen in the lower panels of Fig. 4 (showing *θ* along the trajectories) and Supplementary Fig. S1, showing atmospheric pressure along the trajectories, giving information on adiabatic changes.

### Forecasts

Over the past decade, “ensemble” weather forecasting has become prevalent in operational forecast offices, particularly at the medium to long forecast horizons (~4-14 days into the future). A forecast ensemble contains many models, or “members,” that typically have varied initial states and/or physics parameters. The benefits of this approach are twofold: (1) the mean or median of the ensemble forecasts, typically taken as the most likely outcome, is usually more accurate than any single model and less subject to false alarms; and (2) the members sample the uncertainty space, thus giving users a sense of the range and probability of various outcomes. Statistical post-processing is typically applied to the raw ensemble output to improve the accuracy of both the mean/median, and the probabilities derived from the distribution of ensemble members.

### Subseasonal forecasts

This heatwave was characterized by record-breaking surface temperatures and a slowly propagating blocking high with extreme geopotential anomalies at 500 hPa (z500). Thus, to evaluate the subseasonal to seasonal (S2S) forecasts, we focus on probabilistic forecasts of 2 m air temperature (t2m) anomalies and frequency of blocking^90^ observed over the days between 25 June and 1 July, and compare to those in ERA5 data^84^. Soil moisture analysis was conducted using ERA5-land data^91^.

We use the state-of-the-art ECMWF Integrated Forecasting System (IFS) model, one of 11 models available as part of the S2S prediction project database^92^. The IFS has 51 forecast ensemble members, with 11 reforecast members implemented over the previous 20 years, and a maximum resolution of 0.5°. Forecasts and reforecasts are initialized twice a week. Previous evaluations of ECMWF indicate good performance up to lead times of 7–10 days for extreme cold events^93^, up to 11 days for daily precipitation^94^, up to 15 days for heatwaves^95,96^, and up to 20 days for soil moisture^97^.

To understand the prediction skill of ensemble forecasts for an extreme event we take a probabilistic approach^98^. We investigate the forecast skill in predicting ‘an extreme’, rather than necessarily an extreme of the magnitude observed. We show results for an extreme event defined as one exceeding the 95th percentile, but similar results are obtained for the 90th and 99th percentiles. We identify the 95th percentile threshold for the variable of interest (temporally averaged t2m anomalies or number of blocked days) from 25 June to 1 July across all reforecast years (2001–2020) and all ensemble members. We then calculate the fraction of forecast ensemble members that predicted t2m anomalies or number of blocked days higher than this threshold for 25 June to 1 July 2021. Comparison to the expected value for a climatological forecast (0.05 for the 95th percentile) gives the increased probability of the event as predicted by the ensemble forecast.

The model climatology, required to calculate anomalies, was calculated from the reforecast data using the methodology of Owens and Hewson^99^, averaging data from reforecasts initialized on the same day-of-year as the forecast being analyzed, as well as reforecasts initialized in the days/weeks prior to, and following, the forecast initialization date. This method eliminates systematic biases of the forecasted weather relative to the climatology, and accounts for model drift due to changing lead times. The original methodology has a slight difference in the number of consecutive reforecasts used to calculate the climatology for lead times greater than, or <15 days; for consistency across all lead times considered in this work, we calculate all climatologies using 5 consecutive reforecasts. The climatology is calculated for years 2001–2020. For example, for the forecasts initialized on 14 June 2021, the climatology is calculated from reforecasts initialized on 7, 10, 14, 17, and 21 June from all years from 2001–2020. For consistency between models and reanalysis data, the ERA5 climatology is calculated using years 2001–2020 and smoothed using a 5-day running mean.

Blocking frequency was calculated based on a modified^100^ version of the algorithm proposed by Dunn-Sigouin et al.^101,102^, using geopotential height at 500 hPa (z500). For the algorithm to define a block, a persistent, positive z500 anomaly, spanning at least 10^6^ km^*2*^, must occur in the same region as a reversal of the meridional absolute z500 gradient. Anomalies must be greater than a given threshold calculated using data from all longitudes and latitudes 30–90°N (data normalized by latitude); we use a threshold of 1 standard deviation^100^. In the original method the standard deviation is calculated for a 3-month period centered around the given month; to implement this for the forecasts using a methodology that could be operationalised (i.e., not relying on information from the future), we calculate the standard deviation based on all June values from ensemble members with June initializations across all available years. As the number of ensemble members varies between forecast (2021) and reforecast (2001–2020) years, standard deviations are calculated separately for each year and then averaged. The persistence requirement is considered to be satisfied if the event lasts for at least 5 days.

To compare to ERA5 data, in Fig. 5 the location of the heatwave event in ERA5 is shown in gray contours, as determined by temperature anomalies greater than the 90th percentile and 5 °C, and areas where there is blocking for at least 5 of the 7 days (25 June to 1 July inclusive).

### Climate change context

Analysis of the temperature trends was conducted using the Berkeley Earth temperature anomaly dataset^103^. We calculate annual and JJA (June-August) averages of latitude-weighted monthly mean, and monthly mean daily maximum (TX) temperatures over land for the region 45N–52°N, 119W–123°W, as highlighted in Fig. 1a; results are relatively insensitive to changes in this region definition. We analyze data over the period 1875–2020, the time period for which there was a valid value for every gridbox over the selected region in the Berkeley Earth dataset, and excluding the 2021 heatwave event. We calculate linear fits to approximate the trends, finding a value of 0.10 °C/decade (*p* value 0.4E-9) for annual mean temperatures, 0.07 °C/decade (*p* value 0.3E-5) for JJA mean temperatures, and 0.04 °C/decade (*p* value 0.1) for JJA monthly mean TX.

The PAMIP simulations analyzed are described in detail in Smith et al.^35^, and were designed to isolate the impacts of sea-ice loss and sea surface temperature (SST) changes on atmospheric circulation. The experiments we analyze use fixed SSTs and sea-ice concentration (SIC) as boundary conditions for an atmosphere-only general circulation model. To isolate the impact of sea-ice loss alone on atmospheric circulation patterns, we use simulations with present-day SSTs and pre-industrial Arctic SIC (pdSST-piSIC), present-day SSTs and present-day SIC (pdSST-pdSIC), and present-day SSTs with future Arctic SIC (pdSST-futArcSIC). We further contrast the present-day simulations (pdSST-pdSIC) with those with pre-industrial SSTs and SIC (piSST-piSIC) to understand if changes in SST may have increased the probability of amplified atmospheric waves*.*

There are many available metrics of atmospheric ‘waviness’; to avoid results that may be highly sensitive to the metric selected, we choose two metrics that use different variables on different levels within the atmosphere. The first is the finite-amplitude local wave activity (LWA);^104,105^ we calculate the LWA using geopotential height at 500 hPa (z500), a formulation which has been shown to be strongly connected to persistent weather systems^106^, with the anti-cyclonic LWA (a-LWA) associated with blocking^107^. We analyze differences within the PAMIP simulations in a-LWA. The second metric used is an algorithm to detect recurrent, or quasi-stationary, Rossby waves;^108,109^ this metric, *R*, uses 14-day running mean meridional wind speeds at 250 hPa (v250). When applied to ERA5 reanalysis data^84^ both *R* and *a-LWA* show a positive anomaly over the PNW region during the heatwave event relative to the 1979–2020 climatology for June/July, demonstrating their suitability as metrics for this type of event.

We analyze results from two models from the PAMIP project, the IPSL-CM6A-LR, and CanESM5, downloaded using acccmip6^110^. These models were chosen as the only models with both z500 and v250 at the required daily temporal resolution available on the Earth System Grid at the time of research. These two models cover a wide range of the different model responses of the mid-latitude circulation to sea-ice loss within the PAMIP model ensemble, with the CanESM5 showing a strong reduction in mid-latitude zonal winds, and the IPSL-CM6A-LR a similar, but much weaker response^36^*.* We note that imposing sea-ice loss alone misses other aspects of the AA response to anthropogenic warming^111^, and the response may be sensitive to how or where AA is imposed^112,113^.

### Human health impacts

All information in this section has been taken from other published reports or sources. Excess deaths, heat-attributable deaths, and mortality rates, during and after the heatwave were sourced from British Columbia Centre for Disease Control^48^ and coroners reports for BC^49^, State or County reports for the states of Washington^51^ and Oregon^114^, and local news reporting of preliminary reports for Alberta^50^. Estimates of heat-related mortality can vary between different reports due to different definitions and reporting methods. For example, ‘excess deaths’ can differ from ‘heat-related deaths’, where in the latter, excessive heat has been specifically identified as a contributing factor in the cause of death. Heatwaves can also contribute to deaths that occur in the days or weeks following the event itself, and attributing these deaths to the heatwave can be difficult and time-consuming, and thus estimates for this heatwave may continue to be revised. Non-fatal hospital admission data were from hospitals reporting to the National Syndromic Surveillance Program. The notice from WorksafeBC-advising employers to consider workplace closures during the extreme heat event can be found here: https://www.worksafebc.com/en/about-us/news-events/news-releases/2021/June/worksafebc-advising-employers-to-consider-workplace-closures-during-heat-wave, with local news sources reporting that businesses followed this advice^115^.

### Marine life

Bay mussel (*M. trossulus*) mortality surveys were conducted on a representative intertidal boulder field at the Porteau Cove Marine Park, BC (49.5613°N, −123.2336°W) on 8 July 2021. The odor of rotting marine life was still pronounced at this site even 10–12 days after the peak of the heatwave. Randomly placed 100 cm^2^ quadrats (*n* = 20) were used to determine the number of live and dead mussels. Because mussels gape very soon after death (see Fig. 6b, c) and this survey was conducted 10 days after the heatwave, we assumed that mussels with closed valves were alive, and categorized empty, gaping shells as dead. The mean mortality across quadrats was 73.6 ±  6.2% (mean ± s.e.), with an average of 10.0 ± 1.8 dead mussels per 100 cm^*2*^ quadrat (equivalent to 1000 dead mussels per m^*2*^). The mussel population at this site occurred primarily in a zone 12-meters wide (cross-shore), suggesting that ~1,200,000 mussels died within the ~100 m alongshore extent of this boulder field. Although mussel shells remain attached to the shore via byssus for many weeks after death, we cannot rule out that our surveys slightly underestimated mortality due to shell loss (there was some evidence for this in some quadrats), or overestimated it by including previously dead animals (although the percentage of standing dead mussels is typically low as evidenced by near 100% live mussel cover at microsites in Porteau Cove and elsewhere spared from the heatwave by seaweed canopy or north-facing topographic orientation).

Estimates of barnacle deaths were obtained from random quadrat sampling (100 cm^*2*^ quadrats, *n* = 25) of barnacles (*B. glandula*) at a gently sloping cobble and boulder shore (1001 Steps Park, 49.0306°N, −122.8752°W). Unlike mussels, which gape very soon after death, barnacles can retain their opercular plates for a few weeks after they have died, making mortality more difficult to discern visually. The barnacle mortality surveys reported here were conducted on 25 July 2021, which allowed sufficient time for opercular plates of recently-deceased barnacles to detach and wash away. Barnacles lacking opercular plates were recorded as dead; barnacles with opercular plates held in their natural position were assumed to be alive, which was confirmed by prodding the opercular plates of a subset of these individuals with a pencil to verify the presence of a living animal holding them closed. Sampling revealed 80.6 ± 5.7% mortality (mean ± s.e.), which far exceeds the pre-heatwave background frequency of standing dead measured at this site (7.9 ± 1.2%). The absolute number of excess dead barnacles per 100 cm^2^ quadrat was 54.8 ± 9.4 (equivalent to 5480 per m^2^). Scaling up to the entire barnacle zone at this site (18 m wide in the cross-shore direction) suggests this heatwave killed roughly 10 million barnacles along a single 100 *m* stretch of cobble shoreline. Qualitative surveys of barnacle and mussel mortality at other sites in BC and Washington State suggest that these high rates of mortality were experienced throughout much of Puget Sound and the Strait of Georgia, with lower but still noticeable mortality further north along the BC Central Coast^61^.

Intertidal temperatures during the peak of the heatwave (the afternoon of 28 June 2021 in the vicinity of Vancouver, BC) were recorded with a FLIR E40 camera (Fig. 6a, b). The mortality of various species was documented opportunistically via additional photographs. The images in Fig. 6 were taken at the following locations and dates: (c) Lighthouse Park, West Vancouver, BC, 28 June 2021; (d, g, h) 1001 Steps Park, Surrey, BC, 11 July 2021; (e) Elliot Beach Park, Ladysmith, BC, 10 July 2021; f) Stanley Park, Vancouver, BC, 12 July 2021; and i) Selma Park, BC, 16 July 2021.

### Wildfires

The Fire Weather Index System (FWI), combines components accounting for the effects of both fuel moisture and weather conditions on fire behavior^116^. The system iteratively tracks the effects of the local weather on the forest vegetation moisture content, combining this with wind-affected fire spread rates to yield a dimensionless index value ranging on average from 0 to 30, where higher values represent the potential for extreme fire behavior.

The FWI values shown in Fig. 7 are derived and iteratively tracked using output from a numerical weather prediction (NWP) model. The NWP model used is the Weather Research, and Forecasting model Version 4.1.2 (WRFv4) initialized with the North American Mesoscale (NAM) model with two-way nested domains of 36, 12, and 4 km^117^. All FWI parameters are solved within the 12 km and 4 km domains with output from the 4 km domain shown in Fig. 7. Further details of the WRF configuration and derived FWI values can be found here:

NWP Derived FWI: https://cerodell.github.io/fwf-docs/build/html/index.html

WRF configuration: http://weather.eos.ubc.ca/wxfcst/html-etc/model-metadata/wan00cg-01.html

Observed FWI data for Lytton, BC on 30 June was obtained from Natural Resources Canada’s (NRCan) Canadian Wildfire Information System (CWFIS) FWI datamart (https://cwfis.cfs.nrcan.gc.ca/downloads/fwi_obs/) under Canada’s Open Government Licence (https://open.canada.ca/en/open-government-licence-canada).

Smoke concentrations in Fig. 7 are outputs of ground-level smoke concentrations of particulate matter PM2.5 estimated from the BlueSky smoke forecasting system^118^. The BlueSky model was developed by the United States Forest Service (USFS) AirFire Research Group using inputs of fire information from satellite observations and meteorology to estimate fire emissions, initial smoke-plume rise, and subsequent 3-D smoke dispersion. The University of British Columbia (UBC) Weather Forecast and Research Team (WFRT) runs a version of the BlueSky model operationally (firesmoke.ca) during the wildfire season using satellite hotspot information from the smartfire algorithm and meteorology from the WRFv4 model mentioned above^119^. Hotspots in Fig. 7 are satellite image pixels with high infrared intensity, indicating a heat source and thus used as a proxy for wildfires.

Data on wildfires were obtained from reports published online by the BC Wildfire Service (https://www2.gov.bc.ca/gov/content/safety/wildfire-status/about-bcws) and the Canadian Interagency Forest Fire Centre (https://www.ciffc.ca/). In particular, reports were accessed on the Sparks Lake fire and the McKay Creek fire. Data was also obtained from the CIFFC situational reports, in particular reports from 20 June, and 1, 2, 3, and 11 July. A list of the URLs for these reports is included in the Data Availability statement.

Further details on the role of mid-tropospheric moisture in helping produce thermodynamic conditions conducive for pyrocumulonimbus flammagenitus (CbFg) clouds to form over the burning wildfires can be found in the following references^62,63,120,121^. These CbFg clouds can produce large amounts of lightning, providing ignition for more fires. Combustion of the forest vegetation produces smoke particles and releases additional water vapor into strong thermal updrafts above wildfires^62,122^.

### Agricultural yields

Yield estimates were obtained from data made available by Statistics Canada. Provincial crop yields were obtained directly from Table 32-10-0359-01 “Estimated areas, yield, production, average farm price and total farm value of principal field crops, in metric and imperial units”, 10.25318/3210035901-eng; regional crop yields are from Table 32-10-0002-01 “Estimated areas, yield and production of principal field crops by Small Area Data Regions, in metric and imperial units”, 10.25318/3210000201-eng; provincial fruit yields were calculated from Table 32-10-0364-01 “Area, production and farm gate value of marketed fruits” 10.25318/3210036401-eng; and provincial vegetable yields calculated from Table 32-10-0365-01 “Area, production and farm gate value of marketed vegetables”, 10.25318/3210036501-eng. We analyze yields for the four main food field crops in BC—canola, oats, spring wheat, and barley—and all fruit and vegetables crops in BC for which complete data existed (with no missing years) for the required variables for at least 2007–2021.

For field crops, yields were taken directly from the Statistics Canada data, as total production/area harvested. Fruit yields are not available as a direct variable, and so were calculated from marketed production/cultivated area. We use marketed production instead of total production to increase data availability—total production reporting starts in 2011 and has numerous missing years, compared to marketed production reporting starting in 2002 or 2007 (for different fruit). Similarly, we use cultivated area, not bearing area, to increase the number of years of data available and to include in-field losses, i.e., crops that are not even harvested. Whilst this is not the standard definition of yield (total production/area harvested), repeating the calculations with marketed production/bearing area gives similar conclusions for 2021 anomalies. Vegetable yields are calculated as total production/area planted, and thus, as for fruit, our analysis includes in-field losses. Repeating the analysis using total production/area harvested shows similar conclusions for 2021, although generally, the anomalies are smaller, suggesting larger in-field losses in 2021 relative to other years.

To look at 2021 yield anomalies, deviations from the long-term average were determined by first calculating linear annual yield growth rates for each crop (1980–2020 for field crops; 2002–2020 for fruit and vegetables, with the exception of apricots, cranberries, sweet cherries, Brussel sprouts, pumpkins, and squash and zucchini, all of which are 2007–2020). By fitting a linear yield trend, we are able to account for long-term trends due to technological changes and low-frequency climate variability and change. We use these trends to predict the yield for 2021, and calculate the 2021 yield anomaly as the difference between the reported yield and this prediction. To provide an estimate of the magnitude of the 2021 yield changes relative to typical interannual variability, for each crop we calculate the standard deviation, σ, of the annual yield values after removing the linear trend. 2021 anomalies in units of σ are shown for all evaluated crops in Supplementary Fig. S4a. Supplementary Fig. S4b–d shows time series with linear trends and standard deviations for the BC field, fruit, and vegetable crops with the largest 2021 anomalies.

The NDVI is often used to estimate crop yields^123^ and NDVI values are made publicly available for the agricultural region of Canada from Statistics Canada’s Crop Condition Assessment Program (https://www35.statcan.gc.ca/CCAP/en/index). The NDVI is calculated during the growing season from 1000 m resolution digital data from the National Oceanographic and Atmospheric Administration (NOAA) series of satellites and the 250 m MODIS digital data to monitor the changing vegetation conditions on a 7-day cycle. We download these data for regions designated as “cropland” by the Crop Condition Assessment Program (CCAP), and spatially averaged over the 8 census agricultural regions of British Columbia (https://www150.statcan.gc.ca/n1/en/pub/95-630-x/2017000/pdf/Prov59_CARCD-eng.pdf?st=ENVUsTeF). Boundary files for these regions (as used to show the outlines in Figs. 1 and 2) were obtained from Statistics Canada: https://www12.statcan.gc.ca/census-recensement/2021/geo/sip-pis/boundary-limites/index2021-eng.cfm?year=21.

### Glacier and snow melt

Streamflow data were extracted from the Environment and Climate Change Canada Real-time Hydrometric data website^124^. Snow water equivalent data were taken from the ERA5-Land hourly dataset^91^. Glacier area and location data are from the Randolph Glacier Inventory V6.0^125^, whilst basin areas and outlines are from the Water Survey of Canada^126^. Basin glacier coverage is calculated as the summed area of all glaciers^125^ within a basin^126^ divided by the basin area^124^.

Record streamflow values were identified using the Hydrometric data from Environment Canada (https://wateroffice.ec.gc.ca/mainmenu/real_time_data_index_e.html). Examples of all-time record flow during this extreme heatwave are shown in Supplementary Fig. S7a–c. Supplementary Fig. S7d shows daily streamflow records broken for consecutive days on the Lillooet River near Pemberton, where an evacuation order was issued, although the all-time record was not broken.

During the heatwave event, flood watches and warnings along with high streamflow advisories were issued by the BC River Forecast Centre (http://bcrfc.env.gov.bc.ca/warnings/index.htm) and an evacuation order was issued by the Squamish-Lillooet Regional District. Links to the notices were obtained from the Emergency Info BC twitter account (@EmergencyInfoBC). In the attached data files we include a list of the URLs for the Evacuation order, flood watches, and flood warnings.

### Landslides

Post-wildfire debris flows, for example, those shown in Supplementary Fig. S8, were mapped from field observations and satellite imagery (Sentinel imagery dates 3 August 2021; 28 August 2021; 6 March 2022). Vegetation burn severity data were produced by the BC Ministry of Forests, Lands, Natural Resource Operations and Rural Development^127^ using differenced Normalized Burn Ratio (dNBR) and classified according to the United States Forest Service Burned Area Reflectance Classification (BARC) system^128^. Soil burn severity was also evaluated in the field to verify the classification of low, moderate, and high severity provided by the remotely sensed burn severity data. Field evaluation of burn severity was classified based on the apparent degree of mortality of the vegetative canopy and understory, depth of combusted organic matter in the soil, and presence of water repellency. Shallow (<25 cm) test pits were hand dug to identify the percentage of ground cover burned, ash color and depth, changes to soil structure, depth to unburned roots, and soil water repellency. The test pits were accompanied by observations of the forest type and percentage of tree canopy burned. Soil burn severity corresponded to remotely sensed vegetation burn severity.

## Supplementary information

Supplementary Information

## Data Availability

Data collected and/or analyzed for the Overview, Marine Life, and Agricultural Yields sections are provided in the Source Data files. All other data analyzed in this manuscript are available from the following sources: ERA5 reanalysis data: https://www.ecmwf.int/en/forecasts/datasets/reanalysis-datasets/era5 Canada Meteorological data: https://climate.weather.gc.ca/historical_data/search_historic_data_e.html ECMWF IFS subseasonal forecast data: https://apps.ecmwf.int/datasets/data/s2s-realtime-daily-averaged-ecmf/levtype=sfc/type=cf/ PAMIP data: https://esgf-node.llnl.gov/search/cmip6/ Fire Weather Index: https://cwfis.cfs.nrcan.gc.ca/downloads/fwi_obs/ General wildfire reporting: https://www2.gov.bc.ca/gov/content/safety/wildfire-status/about-bcws; https://www.ciffc.ca Active wildfires information: Sparks Lake wildfire: BC Wildfire Service Wildfires of Note - Sparks Lake (K21001) http://bcfireinfo.for.gov.bc.ca/hprScripts/WildfireNews/OneFire.asp?ID = 811 McKay Creek wildfire: BC Wildfire Service Wildfires of Note - Mckay Creek (K71030) https://bcfireinfo.for.gov.bc.ca/hprScripts/WildfireNews/OneFire.asp?ID = 812 Canadian Interagency Forest Fire Centre Inc. National Fire Situation Report - June 20. https://ciffc.net/en/ciffc/sitrep/2021-06-20 Canadian Interagency Forest Fire Centre Inc. National Fire Situation Report - July 1st. https://ciffc.net/en/ciffc/sitrep/2021-07-01 Canadian Interagency Forest Fire Centre Inc. National Fire Situation Report - July 2nd. https://ciffc.net/en/ciffc/sitrep/2021-07-02 Canadian Interagency Forest Fire Centre Inc. National Fire Situation Report - July 3rd. https://ciffc.net/en/ciffc/sitrep/2021-07-03 Canadian Interagency Forest Fire Centre Inc. National Fire Situation Report - July 11th. https://ciffc.net/en/ciffc/sitrep/2021-07-11 Crop yield data: 10.25318/3210035901-eng; 10.25318/3210000201-eng; 10.25318/3210036401-eng; 10.25318/3210036501-eng Normalized Difference Vegetation Index data: https://www35.statcan.gc.ca/CCAP/en/index Hydrometric data from Environment Canada: https://wateroffice.ec.gc.ca/mainmenu/real_time_data_index_e.html Specific URLs for the reported record streamflow and evacuation/flood orders are listed within the attached data files. Source data are provided with this paper.

## Source data

Source Data

## Code availability

Code to reproduce Figs. 1–3 and S5 is available on GitHub: https://github.com/rhwhite/SupportingInformation/tree/main/June2021heatwave

The NOAA HYSPLIT model used to create Fig. 4 and S1 is available online: https://www.ready.noaa.gov/HYSPLIT_traj.php

Details on the WRF model setup for the Wildfires section and Fig. 7 is available here:

https://cerodell.github.io/fwf-docs/build/html/index.html

http://weather.eos.ubc.ca/wxfcst/html-etc/model-metadata/wan00cg-01.html

Code to reproduce Fig. 8 and S4 and analysis for the *Agricultural Yields section* are available in the attached data file.

Code to reproduce Fig. 9 and S6 and analysis for the *Glacier and Snow Melt* section is available on GitHub: https://github.com/andersonsam/pacific_northwest_heatwave.
