# Supplementary material for: The unprecedented Pacific Northwest heatwave of June 2021
Source: Nat Commun. 2023 Feb 9;14:727. doi: 10.1038/s41467-023-36289-3 (PMC9910268; doi:10.1038/s41467-023-36289-3)
Supplement: Supplementary file 1 — Supplementary Information [file 41467_2023_36289_MOESM1_ESM.pdf]

The Unprecedented Pacific Northwest Heatwave of June 2021: Supplementary Figures

Rachel H. White[1*], Sam Anderson[1], James F. Booth[2,3], Ginni Braich[4], Christina Draeger[1], Cuiyi Fei[1], Christopher Harley[5], Sarah B. Henderson[6,7], Matthias Jakob[1,8], Carie-Ann Lau[8], Lualawi Mareshet Admasu[1], Veeshan Narinesingh[9], Christopher Rodell[1], Eliott Roocroft[1], Kate Weinberger[7] and Greg West[1,10]

[1*]Department of Earth, Ocean and Atmospheric Sciences, University of British Columbia, BC, Canada.
[2]Earth and Atmospheric Science, City College of New York, NY, US.
[3]The Graduate Center, City University of New York, NY, US.
[4]Institute for Resources, Environment and Sustainability, University of British Columbia, BC, Canada.
[5]Department of Zoology, University of British Columbia, BC, Canada.
[6]Environmental Health Services, British Columbia Centre for Disease Control (BCCDC), BC, Canada.
[7]School of Population and Public Health, University of British Columbia, BC, Canada.
[8]BCG Engineering Inc, BC, Canada.
[9]NOAA Geophysical Fluid Dynamics Laboratory, Program in Atmosphere and Ocean Sciences, Princeton University, NJ, US.
[10]BC Hydro, BC, Canada.

*Corresponding author(s). E-mail(s): rwhite@eoas.ubc.ca;

This document contains Supplementary Figures S1-S8.

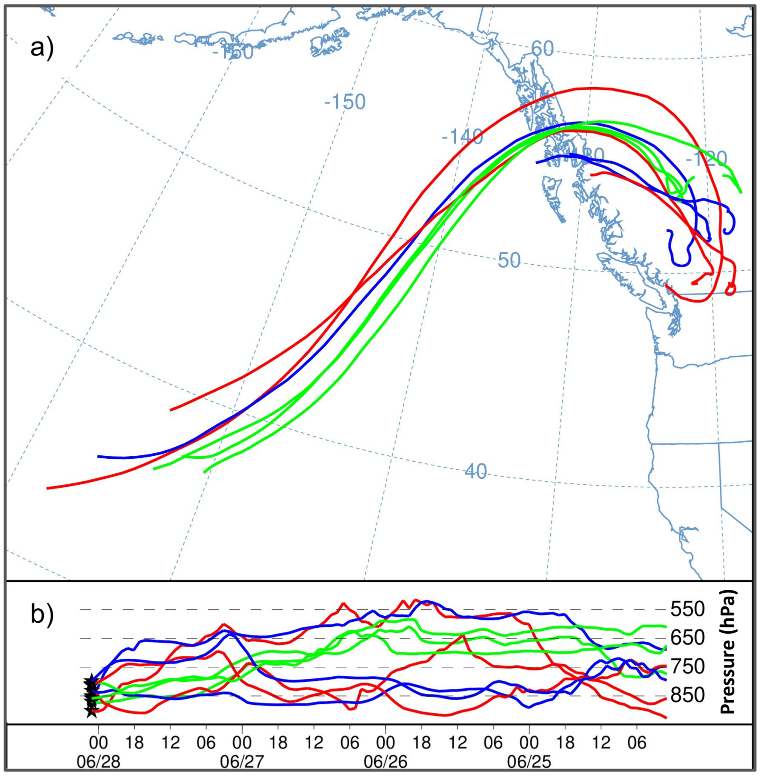

Fig. S1 Trajectories as in Fig. 4, but in a) trajectory paths are plotted without time markers for better readability, especially over BC; and in b) pressure (hPa) is plotted along trajectory paths instead of potential temperature; time is from right to left. 63 similar trajectories, including these 9, were used to calculate temperature changes due to diabatic and adiabatic processes for parcels terminating within the heat anomaly.

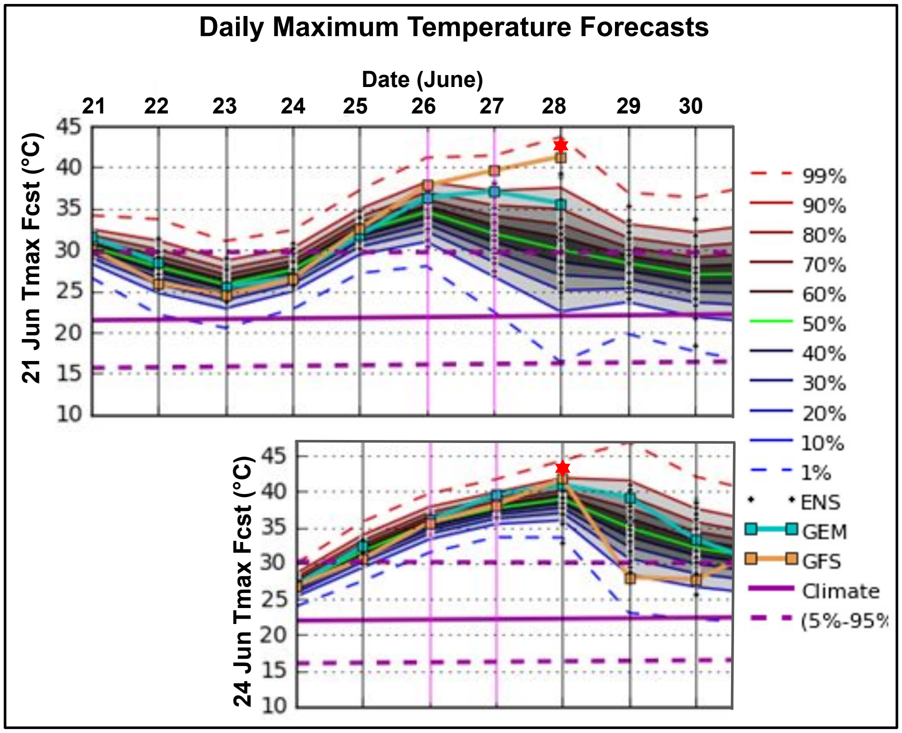

Fig. S2 Ensemble meteogram forecasts for daily maximum temperature, TX, for an example location representative of the BC South Coast region (Abbotsford, BC). Probabilities from the fitted cumulative continuous distribution from the post-processed NAEFS forecasts are shown in the grey shading, with lines every 10th percentile from 10 to 90%; the 1st and 99th probabilities are shown by blue and red dashed lines, respectively. Individual post-processed members are shown with black dots. Two forecasts from deterministic models are also shown—in blue the Canadian Regional Deterministic Prediction System (for forecast days 1 and 2) with the Global Deterministic Prediction System (for forecast days 3-8), denoted 'GEM' in the legend, and in orange the U.S. Global Forecast System (GFS). The 30-yr (1981-2010) climatological 50th percentile (solid magenta), and 5th and 95th percentiles (dashed magenta) are plotted for reference. The red star indicates Abbotsford's hottest observed temperature of the heatwave: 42.9°C on 28 June.

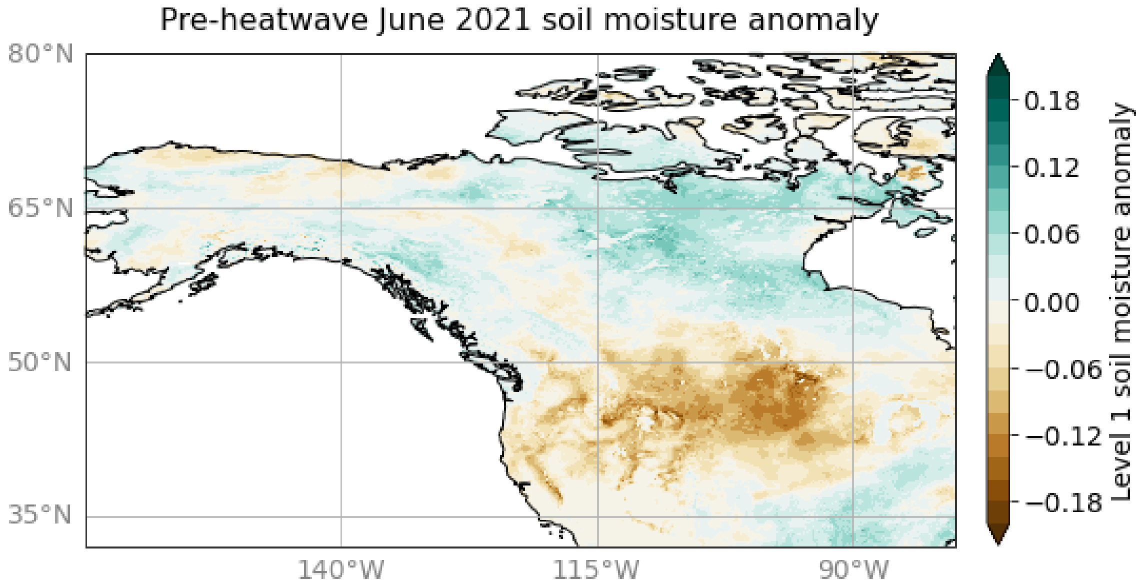

Fig. S3 ERA5 Land level 1 soil moisture anomaly for 1 June 2021 to 24 June 2021, relative to a 1981-2020 climatology. As the unprecedented heatwave began on approximately 25 June 2021, this excludes the impact of the heatwave on the soil moisture.

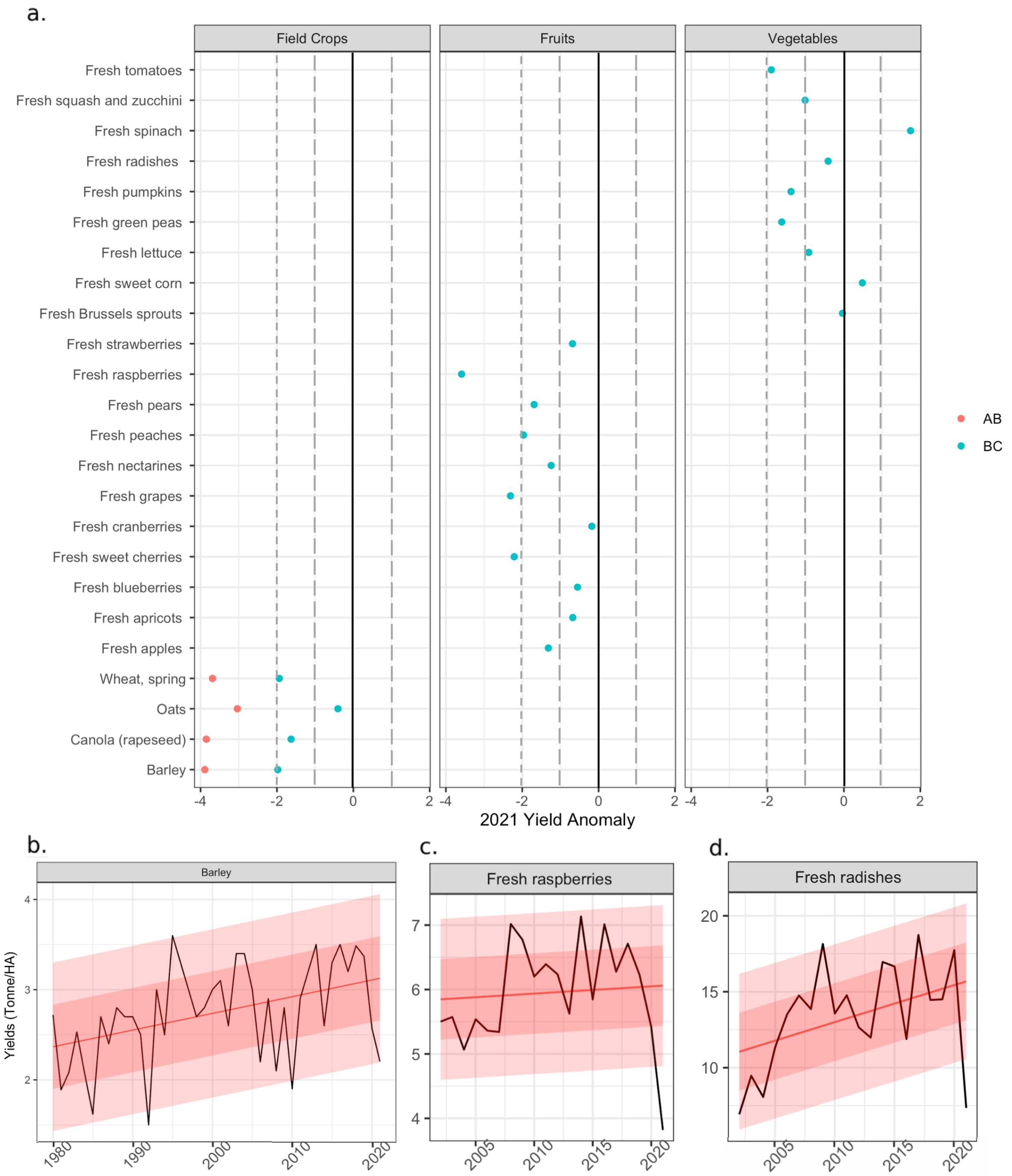

Fig. S4 a) 2021 yield anomalies in standard deviations (σ) for field (left), fruit (centre) and vegetable (right) crops for Alberta (AB, red) and British Columbia (BC; blue). Anomalies are relative to the predicted 2021 value based on a linear trend through all available data (see Methods for more details). -2, +/-1 and 0 are highlighted with dotted, dashed, and solid lines respectively. b-d) estimated yields (black line), fitted linear trend (red line) and +/- 1 and 2σ (shading) for the field (b), fruit (c), and vegetable (d) crops in BC with the largest 2021 reduction in yield.

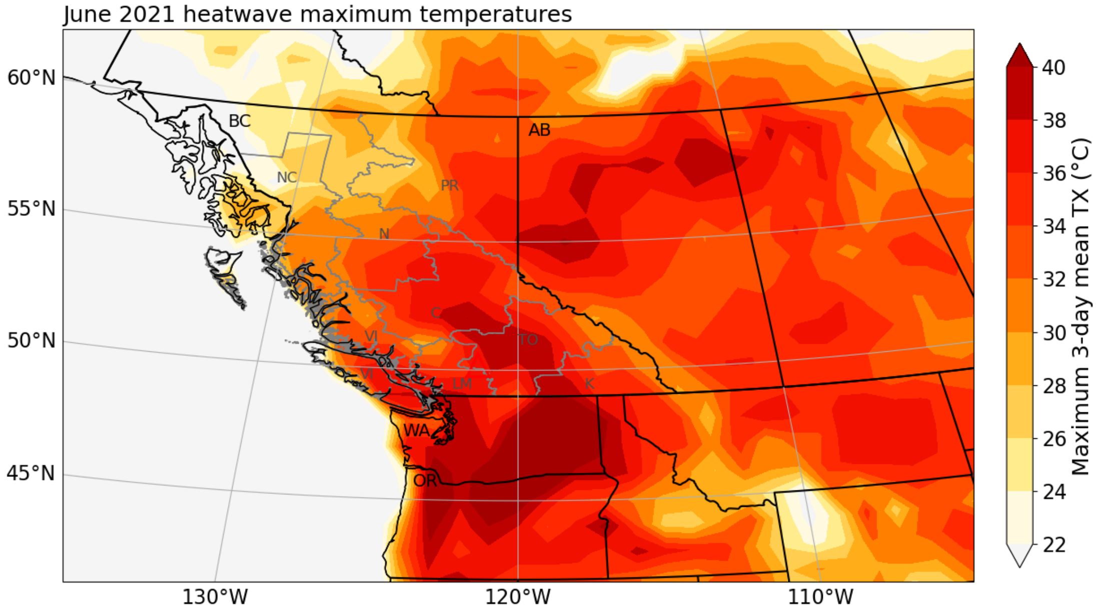

Fig. S5 Maximum 3-day running mean absolute TX between 23 June - 2 July 2021. We use the maximum 3-day mean to emphasize persistent extreme temperatures.

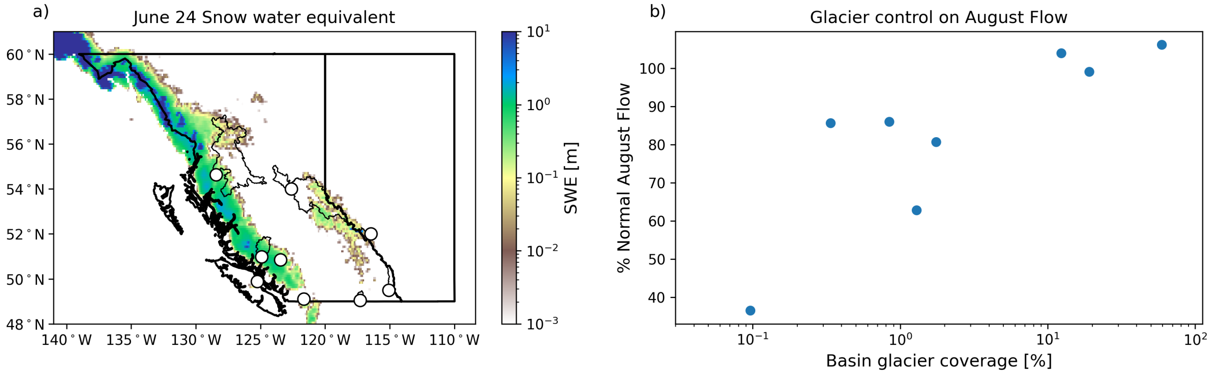

Fig. S6 a) Snow water equivalent (SWE) at the beginning of the heatwave, and the locations of the 9 stream gauges (white circles) used in this study, as well as basin outlines. SWE is concentrated primarily in the Coast and Rocky Mountain ranges. From top to bottom the stream gauge locations are: Skeena River, Fraser River, North Saskatchewan River, Homathko River, Bridge River, Oyster River, Elk River, Chilliwack River (lower left), Salmo River (lower right). b) Total August streamflow in 2021 as a percentage of the 1979-2020 median against basin percentage glacier coverage. Basins with greater glacier coverage experienced less anomalous total August streamflow (closer to or greater than 100% of the median total August streamflow).

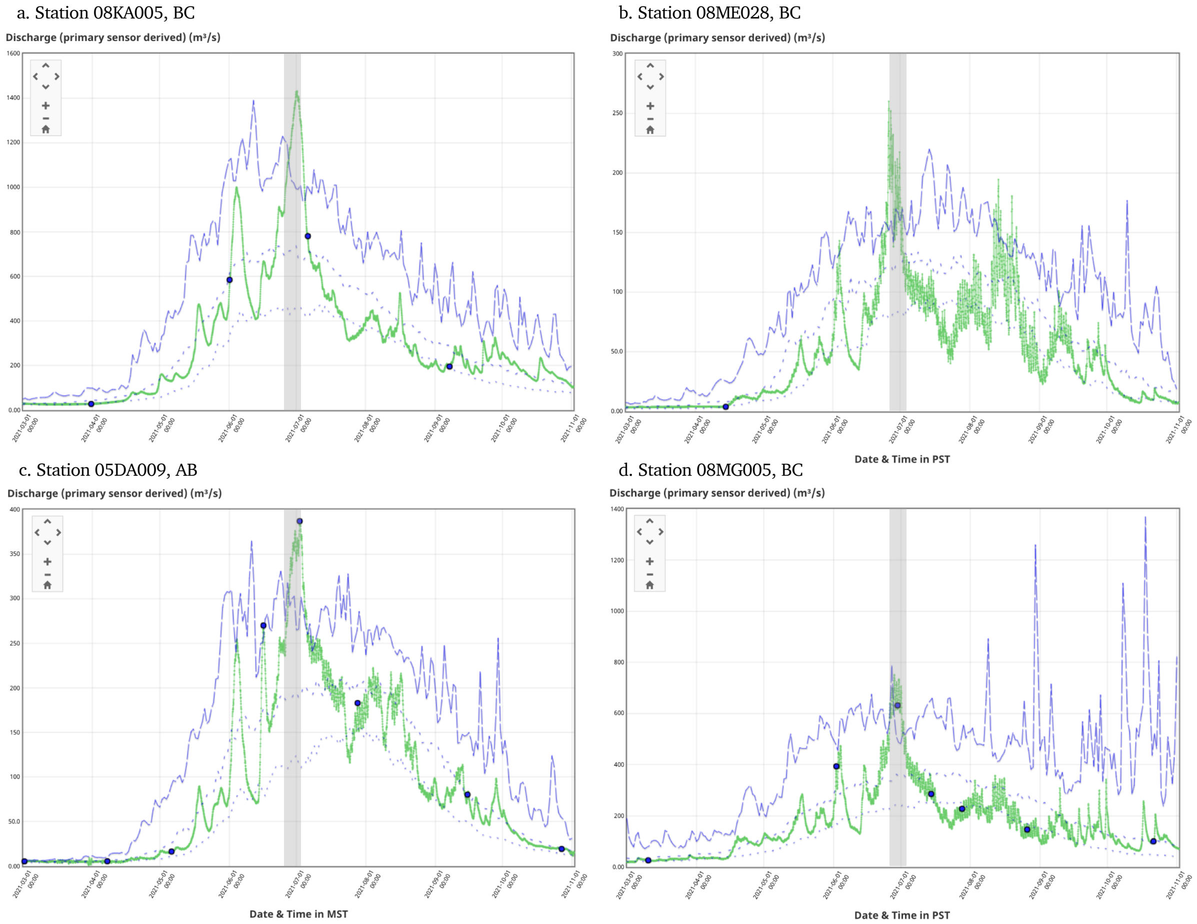

Fig. S7 Hydrometric Data Graphs from Environment and Climate Change Canada real-time data API showing river discharge at selected stations in British Columbia (BC) and Alberta: a) Fraser River at McBride, BC; b) Bridge River above Downton Lake, BC; c) North Saskatchewan River at Whirlpool Point, Alberta; d) Lillooet River near Pemberton, BC. Stations were selected to illustrate all-time records broken during the June 2021 extreme heatwave (a,b,c) and daily records broken (d). Bold green lines show provisional streamflow data for 2021. Blue dotted lines show the upper and lower quartiles for the historical period, and the blue dashed line shows the historical maximum. Grey shading is added to highlight the heatwave period. Record length: a) 70 years, b) 28 years, c) 53 years, d) 105 years. See Methods for links to data. Record streamflow in (b) and (d) were purely snow/glacier-melt driven as there was no or negligible precipitation in these regions during the heatwave. Snow/glacier-melt-driven streamflow in (a) and (c) may have been augmented by precipitation on 1-2 July, exacerbating flooding.

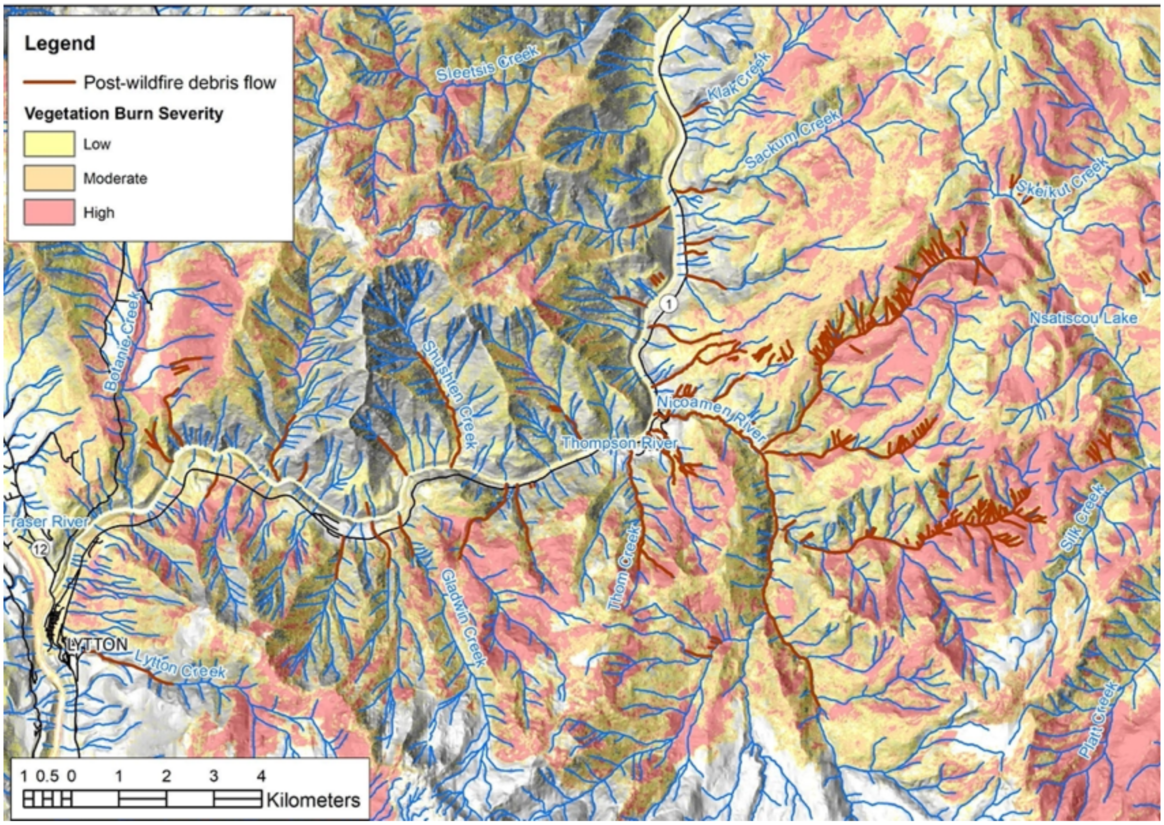

Fig. S8 Distribution of post-wildfire debris flows in the Lytton Creek Fire area and relationship to vegetation burn severity. The highest distribution of post-wildfire debris flows occurred in the Nicoamen River watershed.
