# Supplementary material for: The unprecedented Pacific Northwest heatwave of June 2021
Source: Nat Commun. 2023 Feb 9;14:727. doi: 10.1038/s41467-023-36289-3 (PMC9910268; doi:10.1038/s41467-023-36289-3)
Supplement: Supplementary file 2 — Source Data [file 41467_2023_36289_MOESM2_ESM.zip › June2021_data/glacier_snowmelt/Glacier_snowmelt_streamflow_data_urls.docx]

Unprecedented PNW heatwave of June 2021

Glacier and Snow Melt - data links

Streamflow data were extracted from the Environment and Climate Change Canada Real-time Hydrometric data web site

Data for the streamflow in Supplementary Fig. S6a-d, were obtained from the following links:

<https://wateroffice.ec.gc.ca/report/real_time_e.html?stn=08KA005&mode=Graph&startDate=2021-03-01&endDate=2021-11-01&prm1=47&y1Max=&y1Min=&max1=1&upper1=1&lower1=1&prm2=47&y2Max=&y2Min=>

<https://wateroffice.ec.gc.ca/report/real_time_e.html?stn=08ME028&mode=Graph&startDate=2021-03-01&endDate=2021-11-01&prm1=47&y1Max=&y1Min=&max1=1&upper1=1&lower1=1&prm2=47&y2Max=&y2Min=>

<https://wateroffice.ec.gc.ca/report/real_time_e.html?stn=05DA009&mode=Graph&startDate=2021-03-01&endDate=2021-11-01&prm1=47&y1Max=&y1Min=&max1=1&upper1=1&lower1=1&prm2=47&y2Max=&y2Min=>

<https://wateroffice.ec.gc.ca/report/real_time_e.html?stn=08MG005&mode=Graph&startDate=2021-03-01&endDate=2021-11-01&prm1=47&y1Max=&y1Min=&max1=1&upper1=1&lower1=1&prm2=47&y2Max=&y2Min=>

Evacuation orders, and flood watches and warning are available through the following links:

Squamish-Lillooet Regional District (2021).  EVACUATION ALERT Issued for SLRD Electoral Area C - Pemberton Valley

<https://www.slrd.bc.ca/sites/default/files/pictures/EOC/Evacuation%20ALERT%20%20-%20Electoral%20Area%20C%20-%20Pemberton%20Valley%20Flooding%20-%2026%20Jun%2021_0.pdf>

Squamish-Lillooet Regional District (2021). EVACUATION ORDER Issued for SLRD Electoral Area C - Pemberton Valley

<https://www.slrd.bc.ca/sites/default/files/pictures/EOC/Evacuation%20ORDER%20June%2026%202021.pdf>

Squamish-Lillooet Regional District (2021).  Evacuation order rescind for Electoral Area C - Pemberton Valley

<https://www.slrd.bc.ca/sites/default/files/pdfs/notices/Evacuation%20Order%20RESCIND%20-%20Electoral%20Area%20C%20-%20Pemberton%20Valley%20Flooding_June29_2021.pdf>

Flood Watches (FWT) and Flood Warnings (FWN):

<http://bcrfc.env.gov.bc.ca/warnings/advisories/FWT_2021_0630_1130CentralCoastSouthCoast_upg.pdf>

<http://bcrfc.env.gov.bc.ca/warnings/advisories/FWT_2021_0627_1315Chilcotin_upg.pdf>

<http://bcrfc.env.gov.bc.ca/warnings/advisories/FWN_2021_0630_0930_Chilcotin_upg.pdf>

<http://bcrfc.env.gov.bc.ca/warnings/advisories/FWT_2021_0626_1000_Lillooet_iss.pdf>

<http://bcrfc.env.gov.bc.ca/warnings/advisories/FWT_2021_0630_1330_Morice_upg.pdf>

<http://bcrfc.env.gov.bc.ca/warnings/advisories/FWT_2021_0628_730_UpperColumbia_upg.pdf>

<http://bcrfc.env.gov.bc.ca/warnings/advisories/FWT_2021_0627_1200UpperFraser_iss.pdf>

<http://bcrfc.env.gov.bc.ca/warnings/advisories/FWN_2021_06_29_1300UpperFraser_iss.pdf>
